# Randomized controlled trial of early aerobic exercise following sport-related concussion: Progressive percentage of age-predicted maximal heart rate versus usual care

**DOI:** 10.1371/journal.pone.0276336

**Published:** 2022-12-22

**Authors:** Michael G. Hutchison, Alex P. Di Battista, David W. Lawrence, Kyla Pyndiura, Danielle Corallo, Doug Richards

**Affiliations:** 1 Faculty of Kinesiology and Physical Education, University of Toronto, Toronto, Ontario, Canada; 2 Faculty of Kinesiology & Physical Education, David L. MacIntosh Sport Medicine Clinic, University of Toronto, Toronto, Ontario, Canada; 3 Keenan Research Centre for Biomedical Science of St. Michael’s Hospital, Toronto, Ontario Canada; 4 Centre for Sport-Related Concussion Research, Innovation, and Knowledge, University of Toronto, Toronto, Ontario, Canada; 5 Defence Research and Development Canada, Toronto Research Centre, Toronto, Ontario, Canada; Prince Sattam Bin Abdulaziz University, College of Applied Medical Sciences, SAUDI ARABIA

## Abstract

**Objective:**

To examine the effect of a readily accessible, structured aerobic exercise intervention on days to asymptomatic status and days to medical clearance compared to usual care exercise prescription in a cohort of adolescents and young adults following sport-related concussion (SRC).

**Methods:**

A longitudinal, randomized, non-blinded clinical trial consisting of a structured aerobic exercise protocol (SAEP) group and a usual care exercise prescription (UCEP) group. Participants in the SAEP group underwent an exercise protocol including 8 sessions over 11 days progressing in duration and intensity stepwise based on participants’ age-predicted maximal heart rate. Symptom follow-ups were on days 7, 14, 21, and 28. The primary outcome measures of the study were days to asymptomatic status and days to medical clearance, while the secondary outcome measure was symptom severity on days 7, 14, 21, and 28.

**Results:**

38 participants (SAEP, n = 20; UCEP, n = 19) were recruited and completed all follow-up appointments. Compared to the UCEP group, the SAEP had a faster time to asymptomatic status with 96% posterior probability. In addition, the SAEP group displayed an earlier time to medical clearance with 93% posterior probability. While symptom severity scores did not differ between groups at enrolment (SAEP symptom severity, 30; UCEP, 29), they were subsequently lower in the SAEP group at all assessments throughout the trial with 100% posterior probability.

**Conclusions:**

An aerobic exercise protocol based on percentages of age-predicted maximum heart rate is a safe and effective treatment for reducing symptoms and can be initiated during the first week following SRC.

**Trial registration:**

ClinicalTrials.gov, no. NCT02969824.

## Introduction

A substantial body of research has highlighted the protective effects of aerobic exercise in concussion rehabilitation [1–8]. A recent meta-analysis of eight trials documented a small-to-moderate effect of subthreshold (below symptom exacerbation) aerobic exercise in reducing symptom severity post-concussion [9]. The most recent Concussion in Sport Consensus Statement recommends gradual and progressive activity following an initial period of rest (24–48 hours), with the caveat that activity levels stay below their cognitive and physical symptom-exacerbation thresholds [10]. While specifying aerobic exercise as the activity type is a beneficial update, the dosing, timing, and modalities of exercise within usual care have not yet been elucidated. Hence, current recommendations are still too vague for individual patients and clinicians alike. Providing greater detail to each component of a comprehensive exercise prescription will facilitate an optimal recovery trajectory following injury and prevent protracted recovery. Moreover, there is some evidence that too much activity following sports-related concussion (SRC) may be associated with a recurrence or worsening of symptoms, furthering the need for understanding [11].

Most exercise intervention studies in concussion have required the completion of an exercise tolerance assessment to inform the exercise prescription treatment, leading to the assumption that this resource-intensive evaluation is essential to guide post-injury exercise [3–5, 12]. However, it is unclear if this is necessary; applying general exercise principles (e.g., percentage of age-predicted maximal heart rate) may be similarly effective yet more widely applicable and accessible. Indeed, this research area is still in its infancy, necessitating further investigation into alternative accessible, generalizable methods to inform exercise prescription following concussion.

Several prior randomized studies have employed control groups whereby individuals were advised not to considerably elevate heart rate following concussion [3–5]. However, this does not meet the current standard of care nor usual care practices as per concussion consensus guidelines, potentially limiting the extrinsic validity of the results. The Declaration of Helsinki states that “the benefits, risks, burdens and effectiveness of a new intervention must be tested against those of the best proven intervention(s)” [13]. Given that recent consensus guidelines recommend gradual and progressive exercise following the first 24–48 hours [14], employing a usual care control group encompassing these guidelines is the only valid approach when assessing the effectiveness of any alternative exercise program.

Our earlier work examined the feasibility of an exercise prescription involving a gradual progression of intensity and duration estimated from a percentage of age-predicted maximal heart rate [8]. We found this method to be safe and feasible to administer to symptomatic patients early following sport-related concussion [8]. In addition, we observed faster resolution of symptoms in those who were provided a structured exercise protocol compared to those receiving usual care. However, given the small sample size, replication at a larger scale is needed. Therefore, the purpose of this study was to examine the effect of a readily accessible, structured aerobic exercise intervention on symptoms and time to recovery compared to a usual care exercise prescription group in a cohort of adolescents and young adults following acute SRC. We hypothesized that early structured aerobic exercise guided by percentages of age-predicted maximal heart rate would 1) improve symptoms faster, and 2) lead to an earlier time to medical clearance.

## Materials and methods

### Study design

The current study was a randomized, non-blinded clinical trial consisting of a structured aerobic exercise protocol (SAEP) group and a usual care exercise prescription (UCEP) control group. The study was conducted at an academic sports medicine clinic in Canada, with all participants and parents of minors (aged 13–16 years) having provided free and written informed consent. The trial protocol was registered with clinicaltrials.gov identifier: NCT02969824 and approved by an institutional health sciences research ethics board.

### Participants

Participants were recruited between September 2018 and February 2020 from a sports medicine clinic at a single Canadian academic institute. Eligible participants were required to be between 13–25 years of age with a diagnosis of acute SRC. Concussion diagnosis was determined by a sports medicine physician as per the Concussion in Sport guidelines [15]. Exclusion criteria included a previous concussion within two weeks of the presenting concussion, co-morbid injuries limiting exercise (i.e. musculoskeletal/soft-tissue injuries, vestibular disorders), a pre-existing heart condition, uncontrolled seizure disorders, or a history of medical or neurological conditions that affect cognitive functioning.

### Randomization

Participant randomization was performed using REDCap (Research Electronic Data Capture) electronic data capture tools hosted at the University of Toronto [16, 17]. Eligible participants were randomly assigned to either the SAEP or UCEP groups at the screening visit following informed consent and recording of demographic and injury-related characteristics. An allocation table was then created in REDCap using three strata: sex, initial symptom status, and education level. The research team regularly reminded participants and clinical staff not to discuss their assigned study cohort, and exercise sessions were not performed in the clinic.

### Structured Aerobic Exercise Protocol (SAEP)

Participants were permitted to begin exercise three days post-injury. The exercise protocol consisted of eight, 20-minute sessions over an 11-day period: two days of exercise were followed by one day of rest. Two sessions were completed in-person (the first and fourth exercise session), supervised by a member of the research team at an exercise laboratory using a controlled-power Velotron Dynafit Pro stationary bike manufactured by Racermate Inc. (Seattle, WA, USA). The remainder of the exercise sessions were completed remotely, where participants determined and monitored exercise intensity and heart rate via a Fitbit device. Participants were advised to use a stationary bike to limit head movement and acceleration; however, if participants did not have access to a stationary bike, an elliptical machine, treadmill, or outdoor jogging was permissible.

The protocol progressed in intensity and duration at each session in a stepwise fashion throughout the 11-day period. The intensity of each session was quantified by a calculated target heart rate that progressed from 60% to 75% of the participants’ age-predicted maximal heart rate. For more details on the SAEP, please see the **S1 File**.

### Usual care exercise prescription (UCEP)

Participants allocated to the UCEP group were advised to follow the instructions, prescriptions, and recommendations given to them by the sport medicine physician. Following a brief period of physical and cognitive rest, as suggested in the consensus guidelines, the physician advised participants to increase their activity levels gradually with minimal head movement (predominantly involving a stationary bike) and progressively increase levels of exertion while remaining under the threshold of symptom exacerbation [7]. Subsequently, exercise included a progression of head movements, visual and cognitive burdens, sport-specific activities, and heavy resistance, in that order, all below the symptom exacerbation threshold. For more details on the UCEP, please see the **(S1 File)**. In addition, any diagnosed concomitant neck injuries underwent concurrent rehabilitation. Completion of asymptomatic low-risk simulations of target activities was required prior to medical clearance to return to those activities.

### Trial procedures

Participants were seen by a physician for clinical follow-up across 28 days based on best practices for acute concussion care. All participants completed follow-up visits with a member of the research team on days 7, 14, 21, and 28. At each study visit, participants completed clinical measures including symptom severity rating and C3 Logix testing (balance and cognition).

### Outcomes

The primary outcome measures of the study were days to asymptomatic status and days to medical clearance, while the secondary outcome measure was symptom severity on days 7, 14, 21, and 28.

### Symptoms and subjective rating

Participant symptoms were assessed using the symptom checklist of the SCAT5, which is a 22-item post-concussion symptom scale using a seven-point Likert rating [18]. Symptom severity was calculated by summing the rated score for each symptom up to a maximum of 132. The number of total symptoms was derived by summing the binary presence/absence of each symptom up to a maximum score of 22. In addition, participants rated themselves as a percentage of feeling “normal” (100% = “normal”).

### Medical clearance

Physician determination of full return to a functional domain is individualized and relies on a combination of symptom state, current and premorbid baseline function, and objective assessments. All acute concussions were serially followed until the individual fully returned or was cleared to fully return to sport. Days to medical clearance were calculated for all participants based on days from injury to full return to sport.

### Statistical analysis

Days-to-asymptomatic status (defined as a symptom severity score of seven or lower) [3, 5] from the time of injury to the end of the trial was compared between the UCEP and SAEP groups using exponential (survival) curve modelling; survival analysis was also used to contrast days to medical clearance between groups. In addition, symptom severity scores, total symptom scores, and percent of ‘normal’ were compared between the SAEP and UCEP groups at each of the four assessments via cross-classified linear models. For a more in-depth explanation of the models employed, including prior choices, prior simulations, and posterior predictive checks, please see the **S1 File**.

All models were fit using the Hamiltonian Monte Carlo (HMC) engine Stan [19] via R [20] and the RStudio Integrated Development Environment [21]. The R package ‘rethinking’ [22] was used to interface between RStudio and RStan [23]. Plots were created using the ‘ggplot2’ [24], ‘bayesplot’ [25, 26], and tidybayes [27] packages. Tables were created using the ‘gt’ [28] and ‘gtsummary’ [29] packages. Data and code used in this manuscript can be found in a public github repository **(github link)**.

## Results

### Participant characteristics

**Fig 1:**
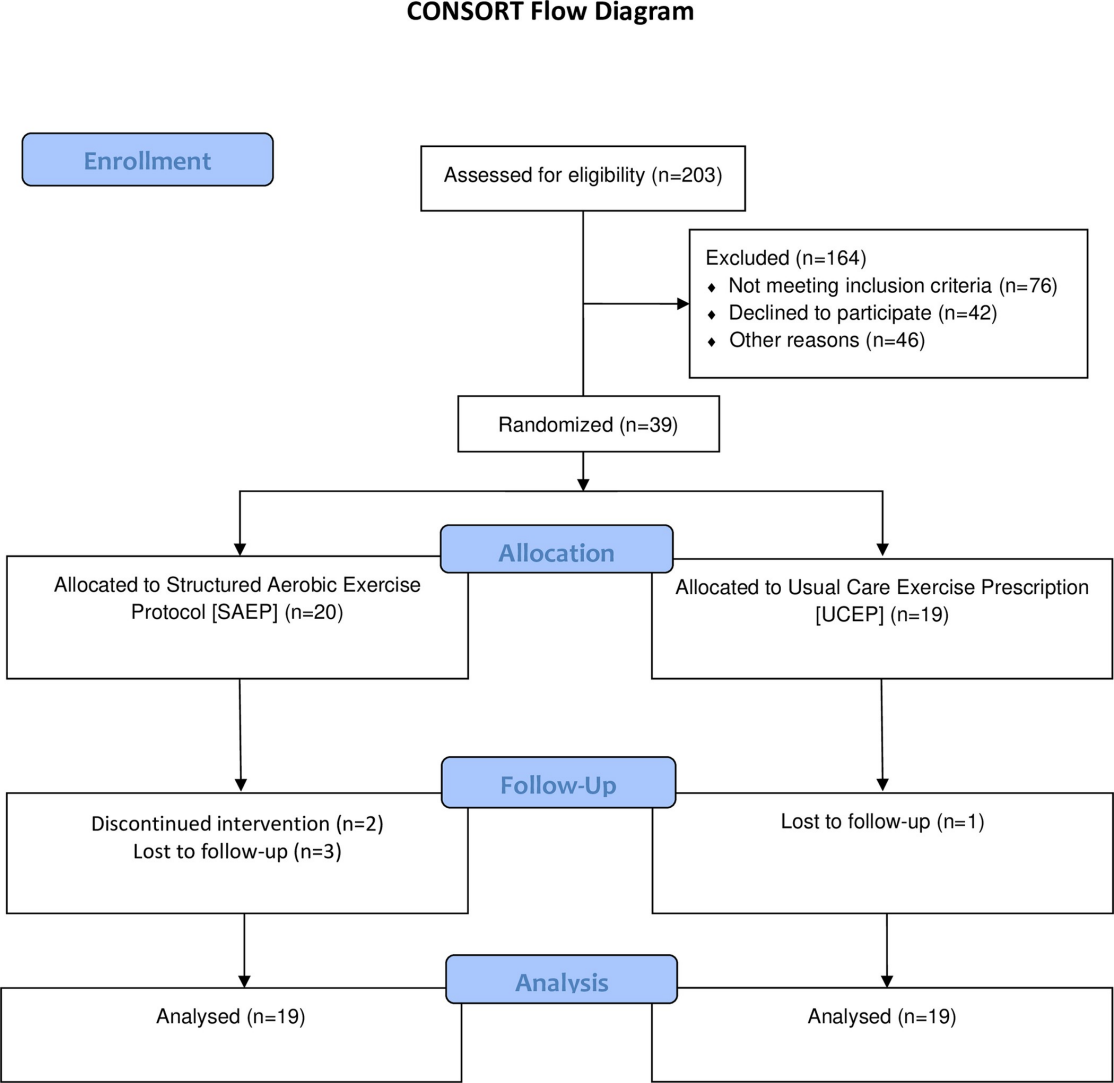
CONSORT flow diagram of participant enrolment. From 203 eligible participants, 39 participants were randomized into either the structured aerobic exercise prescription (SAEP) or usual care exercise prescription (UCEP) groups. Four participants (SAEP = 3, UCEP = 1) were lost to follow-up and thus censored in the statistical analysis. In addition, two participants discontinued the SAEP intervention: one participant at the first exercise session who was removed from the statistical analysis, and one at the third exercise session that was censored in the statistical analysis. Hence, while there were six participants who were either lost to follow-up (n = 4) or discontinued the intervention (n = 2), only one participant was completely removed from the statistical analysis. Therefore, for the statistical analysis of the primary objectives, both groups had 19 participants.

Participant characteristics can be found in Table 1, and subject enrolment data including loss to follow-up and removal can be found in Fig 1. Given that participants in both groups were matched on symptom severity strata, at study enrollment (i.e., randomization) the structured aerobic exercise protocol (SAEP, n = 20) and usual care exercise prescription (UCEP, n = 19) groups had similar symptom severity (median score of 30 vs 29, respectively) and total symptom scores (median 14 vs 13, respectively). Both groups entered the study reporting feeling a median of 65% of their “normal” self. Ages were similar between the SAEP (median = 18, IQR = 16–19) and UCEP groups (median = 21, IQR = 16–22). There were more females in the SAEP group (65% female vs 53% female in UCEP, respectively). Concussion history was more common in the SAEP group, with 58% reporting a prior concussion compared to 45% in the UCEP group.

**Table 1:** Participant characteristics.

Characteristic	SAEP, N = 20	UCEP, N = 19
**Demographics**		
**Age, Median (IQR)**	18 (16–19)	21 (16–22)
**Female Sex, n (%)**	13 (65)	10 (53)
**Education Level, n (%)**		
College/University	12 (60)	12 (63)
EL/MS/HS	8 (40)	7 (37)
**Sport, n (%)**		
Basketball	2 (10)	1 (5.3)
Boxing	1 (5.0)	0 (0)
Cheerleading	1 (5.0)	1 (5.3)
Figure Skating	1 (5.0)	0 (0)
Football	2 (10)	1 (5.3)
Hockey	4 (20)	3 (16)
Other	2 (10)	8 (42)
Rugby	2 (10)	0 (0)
Soccer	3 (15)	3 (16)
Speed Skating	0 (0)	1 (5.3)
Track and Field	1 (5.0)	1 (5.3)
Volleyball	1 (5.0)	0 (0)
**Prior Concussion #, n (%)**		
0	9 (45)	11 (58)
1	6 (30)	4 (21)
2	1 (5.0)	4 (21)
3	3 (15)	0 (0)
4	1 (5.0)	0 (0)
**ADHD, n (%)**	1 (5.0)	2 (11)
**Learning Disability, n (%)**	0 (0)	2 (11)
**Sleep Disorder, n (%)**	1 (5.0)	0 (0)
**Anxiety, n (%)**	3 (15)	3 (16)
**Depression, n (%)**	3 (15)	3 (16)
**Injury Characteristics**		
**LOC, n (%)**		
Yes	1 (5.0)	0 (0)
No	19 (95)	17 (89)
Unsure	0 (0)	2 (11)
**Total Symptoms, Median (IQR)**	14 (8–18)	13 (10–18)
**Symptom Severity, Median (IQR)**	30 (12–50)	29 (16–46)
**Symptom Severity Range, n (%)**		
< 15 (Low)	6 (30)	5 (26)
16–30 (Moderate)	5 (25)	7 (37)
31–50 (High)	4 (20)	2 (11)
> 50 (Very High)	5 (25)	5 (26)
**% of Normal, Median (IQR)**	65 (59–70)	65 (55–74)
**Godin Score, Median (IQR)**	80 (66–91)	73 (49–97)

Data presented as the Median (IQR), or n (%)

UCEP, usual care exercise prescription; SAEP, structured aerobic exercise prescription, IQR, interquartile range; EL, elementary; MS, middle-school; HS, high school; LOC, loss of consciousness; ADHD, attention deficit hyperactivity disorder

### Adverse events & trial safety

No adverse events were reported during the trial. Two participants in the SAEP group failed two consecutive exercises sessions; failure was defined as a symptom severity increase greater than three points during exercise. Specifically, one participant failed both attempts of the first exercise session performed at 60% of their age-predicted maximal heart rate, while the second participant failed both attempts of the third exercise session at 65% of their age-predicted maximal heart rate. The participant that failed at the first exercise session was not included in the statistical analysis, while the participant who failed after the third exercise session was included in the statistical analysis as a censored observation.

### Time to asymptomatic status

**Fig 2:**
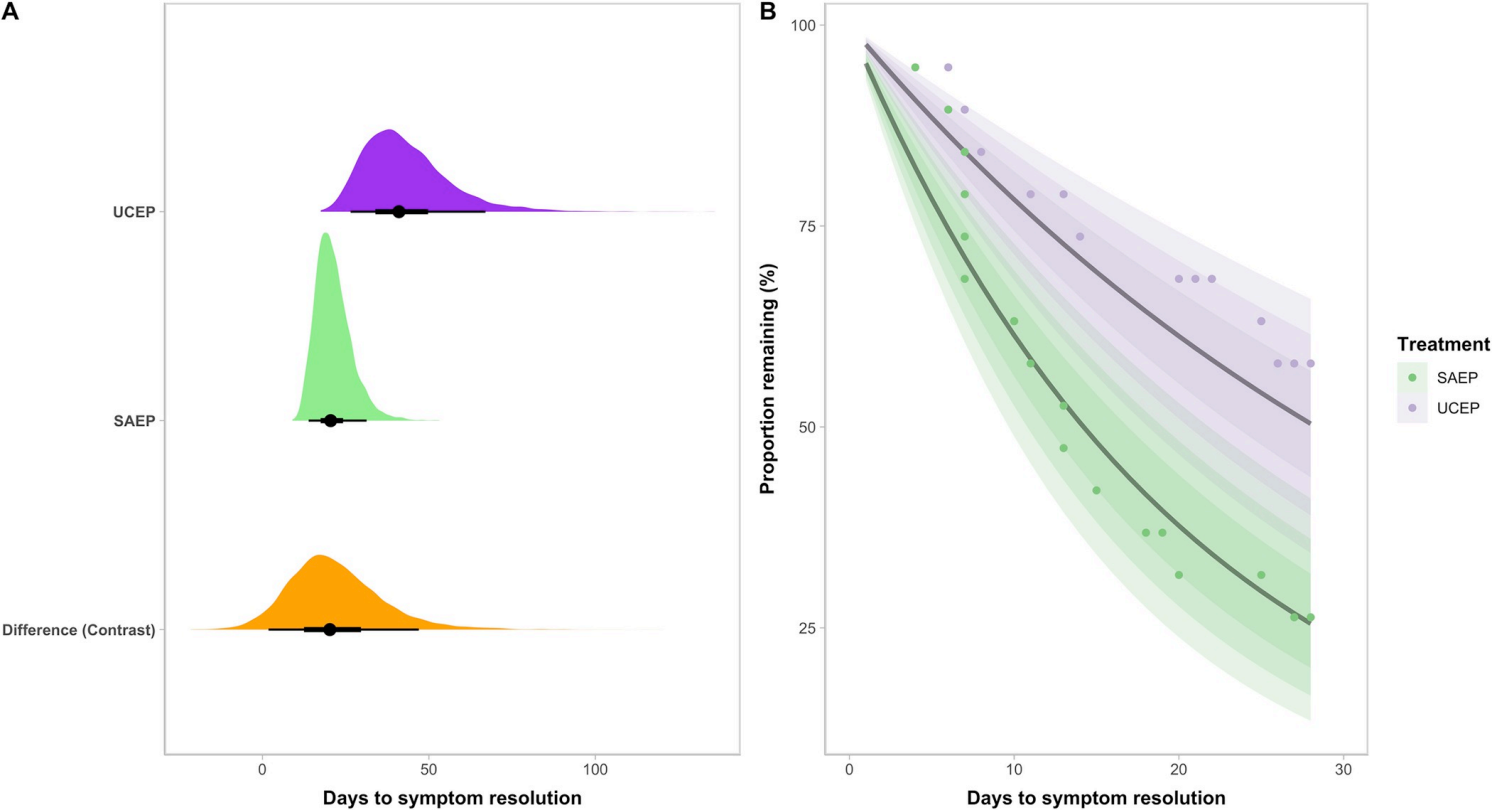
Time to asymptomatic status differs between structured aerobic exercise prescription (SAEP) and usual care exercise prescription (UCEP). **(A)** Histograms display the posterior distribution of credible values representing the days to symptom resolution in the UCEP (purple) and SAEP (green) groups, as well as the difference in days to symptom resolution (orange). **(B)** Posterior samples of the survival curve representing the complementary cumulative probability distribution of the proportion of participants in both groups not yet recovered throughout the trial period (Days 1 through 28). The lines represent the estimated mean for each group, and shaded areas represent the estimated 50%, 70% and 90% Compatibility intervals; the dots represent the raw values.

Visual representation of the survival curve model for days to asymptomatic status can be seen in Fig 2. At ~28 days (the end of the trial), an estimated mean 74% of the participants who underwent SAEP were asymptomatic (90% CI = 59%– 87%) compared to a mean of 50% in those in the UCEP group (90% CI = 34%– 65%). The SAEP group had a 96% posterior probability of becoming asymptomatic faster within the first 28 days following concussion; the 90% CI of the difference in time to asymptomatic status ranged from 2–47 days.

### Time to recovery

Time to recovery was modelled as a survival curve with days to medical clearance as the outcome. The mean estimated time to medical clearance in the SAEP group was 38 days (90% CI = 26–54), compared to 60 days (95% CI = 41–84) in the UCEP group. The SAEP group had a 93% posterior probability of an earlier time to medical clearance; the 90% CI of the estimated difference in time to medical clearance ranged from -3–48 days.

### Symptoms

**Fig 3:**
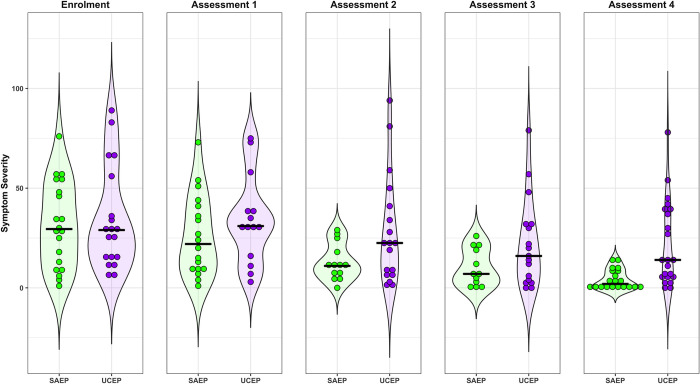
Symptom severity differs between structured aerobic exercise prescription (SAEP) and usual care exercise prescription (UCEP). Dots represent individual symptom severity scores for each subject in the UCEP (purple) and SAEP (green) groups, while the shading represents the mirrored density of the group symptom severity scores. Each panel represents symptom severity scores at one of five time points: Enrolment, then at Assessments 1–4. The black lines represent the group medians.

Descriptive statistics for symptom severity, number of symptoms, and participants’ reported feeling (% of ‘normal’) in both the SAEP and UCEP groups across all four assessments can be seen in **Table 2.** Despite being similar at enrolment, the SAEP group had lower symptom severity scores with 100% posterior probability at each assessment. The estimated 90% compatibility interval (CI) of the difference in symptom scores between groups was 9–21 at assessment one, 7–17 at assessment two, 6–14 at assessment three, and 5–12 at assessment four; the difference was largest at the first assessment (median = 6.5 days from trial enrolment). Please see Fig 3 for a visual comparison of symptom severity scores between SAEP and UCEP groups throughout the trial. Compared to the UCEP group, the SAEP group also displayed a lower total number of symptoms across all four weeks with 99.8% posterior probability, and like symptom severity, the greatest difference was observed at the first assessment where the 90% CI of the difference was 2–7. Perceived percent of “normal” was also consistently lower in the SAEP group at all four assessments with 98.5% posterior probability, although with greater uncertainty than either symptom severity or total symptoms: at each assessment the 90% CI of the difference between groups ranged from 2%– 11.%.

**Table 2:** Participant symptoms across assessments.

Assessment	Symptom Severity		Total Symptoms		% of Normal	
	SAEP, N = 20^*1*^	UCEP, N = 19^*1*^	SAEP, N = 20^*1*^	UCEP, N = 19^*1*^	SAEP, N = 20^*1*^	UCEP, N = 19^*1*^
**Enrolment**	30 (12, 50)	29 (16, 46)	14 (8, 18)	13 (10, 18)	65 (59, 70)	65 (55, 74)
**Assessment 1 (MED = 6.5 days)**	20 (10, 38)	31 (16, 39)	12 (8, 16)	15 (8, 18)	66 (60, 75)	70 (45, 76)
**Assessment 2 (MED = 13 days)**	11 (6, 19)	22 (9, 39)	9 (5, 11)	11 (7, 18)	80 (72, 85)	73 (58, 85)
**Assessment 3 (MED = 20 days)**	6 (0, 12)	18 (4, 32)	4 (0, 9)	10 (4, 15)	90 (81, 94)	78 (66, 90)
**Assessment 4 (MED = 28 days)**	4 (0, 9)	9 (5, 35)	3 (0, 6)	8 (2, 13)	94 (86, 100)	90 (66, 94)

MED, median; SAEP, structured aerobic exercise protocol; UCEP, usual care exercise protocol.

Data presented as the Median (IQR), or n (%); IQR = interquartile range.

Due to loss to follow-up and missing assessments, sample sizes differed across assessments: Enrolment, SAEP = 20 vs, UCEP = 19; assessment 1, SAEP = 19 vs. UCEP = 19; assessment 2, SAEP = 16 vs. UCEP = 19; assessment 3, SAEP = 16 vs. UCEP = 18; assessment 4, SAEP = 14 vs. UCEP = 18.

^*1*^ Median (IQR)

## Discussion

The primary objective of this trial was to compare two groups on their symptom recovery following sport-related concussion: those undergoing structured aerobic exercise guided by percentages of age-predicted maximal heart rate, and those adhering to a usual care exercise program. We observed that participants who underwent an eight-session structured aerobic exercise program (SAEP) over 11 days became asymptomatic faster than those who received usual care exercise prescription (UCEP).

We observed expedited recovery following SAEP on two recovery metrics (days to asymptomatic and medical clearance to RTP) compared to UCEP and found comparatively lower symptom burden in the SAEP group on a weekly basis throughout the trial. Importantly, SAEP led to greater uniformity in recovery versus UCEP. For example, the 90% CI for days to asymptomatic status in the UCEP group was 26–67 days versus 14–31 days in the SAEP group. This equated to ~ 2.5 times less variability in the latter (model SD = 13 vs. 5, respectively). We believe this was in part due to the nature of SAEP treatment–that the frequency, intensity, and duration of exercise sessions was structured uniformly across all participants based on their age-predicted maximum heart rate. Conversely, the relatively looser exercise guidelines given to the UCEP group by their physicians may have resulted in greater variance in the frequency, duration, and intensity of exercise sessions. However, as the UCEP group was treated at a specialty sport medicine clinic, we believe they performed more, and earlier exercise than is typical of patients cared for in community settings. As such, the differences observed between the UCEP and SAEP groups are more striking.

Our results suggest that SAEP does expedite recovery, given that the posterior distribution favoured a comparatively lower time to recovery in the SAEP group an estimated 93% of the time. Direct comparisons of recovery time to prior studies should be interpreted with caution, as there are differences in the operational definitions of clinical recovery. Some recent exercise trials have defined ‘recovered’ as reaching a symptom severity threshold combined with exercise tolerance [3, 5], and others have used return to baseline levels for symptoms, cognition, and balance scores [30]. Our study protocol relied on physician determination of recovery based on aggregate clinical assessments and patient’s tolerance and ability to complete all functional aspects of activity (e.g., social, school, and sport). We believe this definition and estimate of clinical recovery most closely reflects real-world concussion clearance compared to other definitions of “recovery.” In view of this, and as **Fig 3** and **Table 2** illustrate, median symptom severity scores were 11 and 22 on days 7–13 (Assessment two) for the SAEP and UCEP, respectively. These findings are well aligned with other studies in a clinical setting [7] or other Canadian multidisciplinary centres specializing in concussion [31].

It is difficult to compare our results to previous trials looking at the effects of subthreshold exercise on recovery following concussion due to the differences in both population and methodology [3, 32]. Prior studies have focused specifically on a pediatric/adolescent population, used a ‘stretching’ control comparison, and have based their exercise protocol on an initial exercise assessment test [3, 32]. The present study had a comparatively older study sample population and employed a usual care control group. Regarding the latter, we chose this comparator for improved generalizability, as we sought to understand the effect of exercise compared to the standard of care in real-world clinical settings. Finally, the current study used a relatively more resource-friendly protocol that does not require an initial screening test to determine exercise intensity. We caution against comparing the effects among trials because prior studies have used frequentist statistical methods based on significance testing, the derivation of point estimates, and without the incorporation of *a priori* knowledge. The current study utilized a robust Bayesian framework that modeled the complete posterior distribution rather than the derivation of point estimates and did not employ a significance testing framework.

While it is unclear why the SAEP group recovered quicker than the UCEP group in our study, there are various possible underlying causes, and it is likely that the effects of SAEP are multifactorial. It is believed that concussion can cause alterations to energy metabolism in the brain [33], cerebral blood flow [34, 35], and inflammation [36–38]. Hence, it is plausible that exercise may provide some benefit in alleviating dysfunction to one or more of these processes [39–42]. In addition, exercise can have positive psychological effects which may contribute to perceived symptom improvement in participants [43, 44]. However, as a rigorous quantitation of exercise dosing was not performed in the current study, it is not possible to do more than hypothesize on the potential biological/psychological effects of aerobic exercise on recovery following concussion; this remains an important and open question.

There were no adverse effects resulting in the removal of individuals from the exercise intervention. These results are aligned with other trials providing individualized exercise prescription following treadmill assessment [1, 3–5, 45]. Two individuals had temporary exacerbation of symptoms during an exercise session. Current best practice to manage symptom exacerbation following exercise in the acute period is largely determined by the severity and duration of symptom exacerbation, the specific symptom(s) that were provoked, and the type of activity that provoked the symptoms. However, recommendations can include reattempting the same activity after a brief interval period, reducing the intensity or duration of the activity, and/or modifying or substituting the type of activity. Screening for any comorbid confounding medical conditions may also be considered [46]. Future studies may consider greater allowance for temporary changes in symptoms during physical exercise sessions or additional measures of exercise intolerance. Collectively, our results expand on our previous study in a smaller sample which showed the feasibility of a structured aerobic exercise protocol [8], and confirms the safety and efficacy of prescribing early aerobic exercise post-SRC based on percentage of maximum heart rate, progressing in a stepwise manner.

### Limitations

Due to the ongoing COVID-19 pandemic, the trial had to be stopped before reaching our intended sample target; the University clinic closed, and in-person research was prohibited as of March 2020. At the onset of the trial, power analysis yielded a target of approximately 50 participants to identify a mean seven-day reduction in time to asymptomatic status with a moderate effect. Our sample of 39 participants fell short of this sample target, yet at the time of trial stoppage we had identified a much larger reduction and effect in both days to asymptomatic status and time to medical clearance than originally hypothesized. Furthermore, implementing a Bayesian analytical approach was well suited for our modest sample size, and allowed for conservative posterior estimates by using regularizing priors. While a larger sample size would have reduced uncertainty, improved the precision in our parameter estimates, and allowed for estimation of the interacting effects of sex and concussion history, posterior predictive model checks showed that our models mapped well onto the raw data (**Fig 2 and S1 File**). Hence, we feel that the results of this trial are both informative and reproducible. Participants were not blinded to treatment, and thus intervention bias is possible. The SAEP group received two in-person supervised sessions as well as remote supervision and reminders for exercise. These sessions may have accounted for some of the differences between groups by increasing the confidence of study participants in their treatment prescription or ensuring adherence to study procedures [47]. However, participants in both groups had access to interventions, academic accommodations, physical therapy, and pharmacological options associated with sleep, headache, or affective issues, consistent with current guidelines. Finally, the results may not generalize to younger children or older adults, as well as other mechanisms of injury (e.g., assaults, motor vehicle collisions, workplace injuries); trials are warranted to evaluate the utility of structured aerobic exercise across a range of populations and injury mechanisms.

## Conclusion

A structured aerobic exercise intervention following concussion leads to faster symptom recovery and reduces time to clinical recovery compared to usual care. This is the first study to show that an aerobic exercise protocol administered during the first week following SRC and based on percentages of age-predicted maximum heart rate, is a safe and effective treatment for reducing symptoms. The data provide evidence of an early intervention protocol that requires few resources and expertise in exercise prescription.

## Supporting information

S1 File Supplementary methods. Full protocols for the Structured Aerobic Exercise and Usual Care Exercise groups, as well as expanded statistical analysis encompassing model form, prior selection, and data transformations.
(DOCX)

S2 File Consort checklist. Consort checklist.
(DOC)

S3 File Study protocol. Study protocol.
(DOCX)

S1 Fig Posterior predictive check for days to medical clearance. Exponential model for days to return to play overlaid on the complementary cumulative distribution of the raw data.
(TIF)

S2 Fig Prior simulations for exponential models. Prior simulations for exponential models evaluating days to asymptomatic status and days to medical clearance in the structured aerobic exercise prescription (SAEP) vs. usual care exercise prescription (UCEP) groups using a gaussian prior of the form α ~ normal(3.15, 0.6). Simulations results yielded an average time to asymptomatic status/return to play of ~28 days in both the SAEP and UCEP groups, with a standard deviation (SD) of ~18 days, and 90% compatibility interval (CI) of ~ 8.5–65 days. The mean difference between groups (contrast) was ~0, with a SD of ~25 days and 90% CI of ~ -40–40 days.
(TIF)

S3 Fig Prior simulations for symptom comparisons over time. Histograms showing prior simulations for linear regression models evaluating A) Symptom Severity, B) Total Symptoms, and C) “% of Normal” at each of the four assessments of the trial (Assessments 1–4), as well as prior simulation contrasts at each session for the structured aerobic exercise prescription (SAEP) and usual care exercise prescription (UCEP) groups.
(TIF)

S4 Fig MCMC traceplots. Traceplots for exponential survival curve models: (A) days to asymptomatic status, and (B) days to medical clearance. Plots are stratified by varying intercepts for usual care exercise prescription (UCEP) (a[1]) and structured aerobic exercise prescription groups (SAEP) (a[2]). Four chains were run at 20000 iterations per chain. All Gelman-Rubin values were <1.01.
(PDF)

## References

[1] Baker JG, Willer BS, Leddy JJ. Integrating Neuropsychology Services in a Multidisciplinary Concussion Clinic. J Head Trauma Rehabil. 2019;34(6):419–24. Epub 2019/11/07. doi: 10.1097/HTR.0000000000000541 . 31688378

[2] Leddy J, Hinds A, Sirica D, Willer B. The Role of Controlled Exercise in Concussion Management. PM R. 2016;8(3 Suppl):S91–S100. Epub 2016/03/15. doi: 10.1016/j.pmrj.2015.10.017 . 26972272

[3] Leddy JJ, Haider MN, Ellis MJ, Mannix R, Darling SR, Freitas MS, et al. Early Subthreshold Aerobic Exercise for Sport-Related Concussion: A Randomized Clinical Trial. JAMA Pediatr. 2019;173(4):319–25. doi: 10.1001/jamapediatrics.2018.4397 ; PubMed Central PMCID: PMC6450274. 30715132 PMC6450274

[4] Leddy JJ, Haider MN, Hinds AL, Darling S, Willer BS. A Preliminary Study of the Effect of Early Aerobic Exercise Treatment for Sport-Related Concussion in Males. Clin J Sport Med. 2019;29(5):353–60. Epub 2018/09/22. doi: 10.1097/JSM.0000000000000663 ; PubMed Central PMCID: PMC6424660. 30239422 PMC6424660

[5] Leddy JJ, Master CL, Mannix R, Wiebe DJ, Grady MF, Meehan WP, et al. Early targeted heart rate aerobic exercise versus placebo stretching for sport-related concussion in adolescents: a randomised controlled trial. Lancet Child Adolesc Health. 2021;5(11):792–9. Epub 20211001. doi: 10.1016/S2352-4642(21)00267-4 . 34600629

[6] Willer BS, Haider MN, Bezherano I, Wilber CG, Mannix R, Kozlowski K, et al. Comparison of Rest to Aerobic Exercise and Placebo-like Treatment of Acute Sport-Related Concussion in Male and Female Adolescents. Arch Phys Med Rehabil. 2019. Epub 2019/08/05. doi: 10.1016/j.apmr.2019.07.003 . 31377190 PMC6879855

[7] Lawrence DW, Richards D, Comper P, Hutchison MG. Earlier time to aerobic exercise is associated with faster recovery following acute sport concussion. PLoS One. 2018;13(4):e0196062. Epub 2018/04/19. doi: 10.1371/journal.pone.0196062 ; PubMed Central PMCID: PMC5905975. 29668716 PMC5905975

[8] Micay R, Richards D, Hutchison MG. Feasibility of a postacute structured aerobic exercise intervention following sport concussion in symptomatic adolescents: a randomised controlled study. BMJ Open Sport Exerc Med. 2018;4(1):e000404. Epub 2018/07/19. doi: 10.1136/bmjsem-2018-000404 ; PubMed Central PMCID: PMC6045733. 30018795 PMC6045733

[9] Reid SA, Farbenblum J, McLeod S. Do physical interventions improve outcomes following concussion: a systematic review and meta-analysis? Br J Sports Med. 2021. Epub 2021/10/02. doi: 10.1136/bjsports-2020-103470 . 34593371

[10] McCrory P, Meeuwisse W, Dvorak J, Aubry M, Bailes J, Broglio S, et al. Consensus statement on concussion in sport-the 5th international conference on concussion in sport held in Berlin, October 2016. Brit J Sport Med. 2017;51(11):838–47. doi: 10.1136/bjsports-2017-097699 WOS:000402416500003. 28446457

[11] Carson JD, Lawrence DW, Kraft SA, Garel A, Snow CL, Chatterjee A, et al. Premature return to play and return to learn after a sport-related concussion: physician’s chart review. Can Fam Physician. 2014;60(6):e310, e2–5. Epub 2014/06/14. doi: 10.1136/bjsports-2012-092132 ; PubMed Central PMCID: PMC4055342. 24925965 PMC4055342

[12] Haider MN, Johnson SL, Mannix R, Macfarlane AJ, Constantino D, Johnson BD, et al. The Buffalo Concussion Bike Test for Concussion Assessment in Adolescents. Sports Health. 2019;11(6):492–7. Epub 2019/09/06. doi: 10.1177/1941738119870189 ; PubMed Central PMCID: PMC6822206. 31486715 PMC6822206

[13] WMA. Declaration of Helsinki—ethical principles of medical research involving human subjects: World Medical Association; 2013 [cited 2021 November 19].

[14] Thompson BT, Schoenfeld D. Usual care as the control group in clinical trials of nonpharmacologic interventions. Proc Am Thorac Soc. 2007;4(7):577–82. Epub 2007/09/20. doi: 10.1513/pats.200706-072JK ; PubMed Central PMCID: PMC2647648. 17878473 PMC2647648

[15] McCrory P, Meeuwisse W, Aubry M, Cantu B, Dvořák J, Echemendia R, et al. Consensus statement on concussion in sport: the 4th International Conference on Concussion in Sport held in Zurich, November 2012. British Journal of Sports Medicine. 2013;47(5):250–8. doi: 10.1136/bjsports-2013-092313 23479479

[16] Harris PA, Taylor R, Minor BL, Elliott V, Fernandez M, O’Neal L, et al. The REDCap consortium: Building an international community of software platform partners. J Biomed Inform. 2019;95:103208. Epub 2019/05/13. doi: 10.1016/j.jbi.2019.103208 . 31078660 PMC7254481

[17] Harris PA, Taylor R, Thielke R, Payne J, Gonzalez N, Conde JG. Research electronic data capture (REDCap)—a metadata-driven methodology and workflow process for providing translational research informatics support. J Biomed Inform. 2009;42(2):377–81. Epub 2008/10/22. doi: 10.1016/j.jbi.2008.08.010 ; PubMed Central PMCID: PMC2700030. 18929686 PMC2700030

[18] Echemendia RJ, Meeuwisse W, McCrory P, Davis GA, Putukian M, Leddy J, et al. The Sport Concussion Assessment Tool 5th Edition (SCAT5): Background and rationale. Br J Sports Med. 2017;51(11):848–50. Epub 2017/04/28. doi: 10.1136/bjsports-2017-097506 . 28446453

[19] Stan Development Team. Stan Modelling Language 2021. 2.27:[Available from: https://mc-stan.org.

[20] R Development Core Team. R: A language and environment for statistical computing Vienna, Austria: R Foundation for Statistical Computing; 2021. 4.1.0:[Available from: https://www.R-project.org/.

[21] RStudio Team. RStudio: Integrated Development Environment for R. Boston, MA: PBC; 2021. 1.4.1717:[Available from: http://www.rstudio.com/.

[22] McElreath R. rethinking: Statistical Rethinking book package 2020. R package version 2.13:[

[23] Stan Development Team. RStan: the R interface to Stan. 2021. R package version 2.21.2:[Available from: http://mc-stan.org/.

[24] Wickham H. ggplot2: Elegant Graphics for Data Analysis New York: Springer-Verlag; 2016. Available from: https://ggplot2.tidyverse.org.

[25] Gabry J, Mahr T. bayesplot: Plotting for Bayesian Models. 2021. R package version 1.8.1:[Available from: https://mc-stan.org/bayesplot/.

[26] Gabry J, Simpson D, Vehtari A, Betancourt M, Gelman A. Visualization in Bayesian workflow. Journal of the Royal Statistical Society: Series A (Statistics in Society). 2019;182(2):389–402. doi: 10.1111/rssa.12378

[27] Kay M. _tidybayes: Tidy Data and Geoms for Bayesian Models_. 2021. R package version 3.0.0:[Available from: http://mjskay.github.io/tidybayes/.

[28] Iannone R, Cheng J, Schloerke B. gt: Easily Create Presentation-Ready Display Tables. 2021. R package version 0.3.0:[Available from: https://CRAN.R-project.org/package=gt.

[29] Sjoberg DD, Curry M, Hannum M, Larmarange J, Whiting K, Zabor EC. gtsummary: Presentation-Ready Data Summary and Analytic Result Tables 2021. R package version 1.4.1:[Available from: https://CRAN.R-project.org/package=gtsummary.

[30] Maerlender A, Rieman W, Lichtenstein J, Condiracci C. Programmed Physical Exertion in Recovery From Sports-Related Concussion: A Randomized Pilot Study. Dev Neuropsychol. 2015;40(5):273–8. Epub 20150731. doi: 10.1080/87565641.2015.1067706 . 26230745

[31] Ellis MJ, Ritchie LJ, McDonald PJ, Cordingley D, Reimer K, Nijjar S, et al. Multidisciplinary Management of Pediatric Sports-Related Concussion. Can J Neurol Sci. 2017;44(1):24–34. Epub 20161024. doi: 10.1017/cjn.2016.312 . 27772532

[32] Leddy JJ, Master CL, Mannix R, Wiebe DJ, Grady MF, Meehan WP, et al. Early targeted heart rate aerobic exercise versus placebo stretching for sport-related concussion in adolescents: a randomised controlled trial. The Lancet Child & Adolescent Health. 2021. doi: 10.1016/S2352-4642(21)00267-4 34600629

[33] Giza CC, Hovda DA. The new neurometabolic cascade of concussion. Neurosurgery. 2014;75 Suppl 4:S24-33. Epub 2014/09/19. doi: 10.1227/NEU.0000000000000505 ; PubMed Central PMCID: PMC4479139. 25232881 PMC4479139

[34] Churchill NW, Hutchison MG, Graham SJ, Schweizer TA. Evaluating Cerebrovascular Reactivity during the Early Symptomatic Phase of Sport Concussion. J Neurotrauma. 2019;36(10):1518–25. Epub 2018/11/20. doi: 10.1089/neu.2018.6024 . 30451069

[35] Churchill NW, Hutchison MG, Graham SJ, Schweizer TA. Symptom correlates of cerebral blood flow following acute concussion. Neuroimage Clin. 2017;16:234–9. Epub 2017/08/11. doi: 10.1016/j.nicl.2017.07.019 ; PubMed Central PMCID: PMC5545814. 28794982 PMC5545814

[36] Di Battista AP, Churchill N, Rhind SG, Richards D, Hutchison MG. Evidence of a distinct peripheral inflammatory profile in sport-related concussion. J Neuroinflammation. 2019;16(1):17. Epub 2019/01/28. doi: 10.1186/s12974-019-1402-y ; PubMed Central PMCID: PMC6347801. 30684956 PMC6347801

[37] Di Battista AP, Churchill N, Schweizer TA, Rhind SG, Richards D, Baker AJ, et al. Blood biomarkers are associated with brain function and blood flow following sport concussion. J Neuroimmunol. 2018;319:1–8. Epub 2018/04/25. doi: 10.1016/j.jneuroim.2018.03.002 . 29685283

[38] Churchill NW, Di Battista AP, Rhind SG, Richards D, Schweizer TA, Hutchison MG. Cerebral blood flow is associated with matrix metalloproteinase levels during the early symptomatic phase of concussion. PLoS One. 2021;16(11):e0253134. Epub 20211102. doi: 10.1371/journal.pone.0253134 ; PubMed Central PMCID: PMC8562781. 34727098 PMC8562781

[39] Cotman CW, Berchtold NC, Christie LA. Exercise builds brain health: key roles of growth factor cascades and inflammation. Trends Neurosci. 2007;30(9):464–72. Epub 20070831. doi: 10.1016/j.tins.2007.06.011 . 17765329

[40] Rothman SM, Griffioen KJ, Wan R, Mattson MP. Brain‐derived neurotrophic factor as a regulator of systemic and brain energy metabolism and cardiovascular health. Annals of the New York Academy of Sciences. 2012;1264(1):49–63. doi: 10.1111/j.1749-6632.2012.06525.x 22548651 PMC3411899

[41] Kleinloog JPD, Mensink RP, Ivanov D, Adam JJ, Uludag K, Joris PJ. Aerobic Exercise Training Improves Cerebral Blood Flow and Executive Function: A Randomized, Controlled Cross-Over Trial in Sedentary Older Men. Front Aging Neurosci. 2019;11:333. Epub 20191204. doi: 10.3389/fnagi.2019.00333 ; PubMed Central PMCID: PMC6904365. 31866855 PMC6904365

[42] Murrell CJ, Cotter JD, Thomas KN, Lucas SJ, Williams MJ, Ainslie PN. Cerebral blood flow and cerebrovascular reactivity at rest and during sub-maximal exercise: effect of age and 12-week exercise training. Age. 2013;35(3):905–20. doi: 10.1007/s11357-012-9414-x 22669592 PMC3636405

[43] DiLorenzo TM, Bargman EP, Stucky-Ropp R, Brassington GS, Frensch PA, LaFontaine T. Long-term effects of aerobic exercise on psychological outcomes. Prev Med. 1999;28(1):75–85. doi: 10.1006/pmed.1998.0385 . 9973590

[44] Gondoh Y, Sensui H, Kinomura S, Fukuda H, Fujimoto T, Masud M, et al. Effects of aerobic exercise training on brain structure and psychological well-being in young adults. J Sports Med Phys Fitness. 2009;49(2):129–35. . 19528889

[45] Haider MN, Leddy JJ, Wilber CG, Viera KB, Bezherano I, Wilkins KJ, et al. The Predictive Capacity of the Buffalo Concussion Treadmill Test After Sport-Related Concussion in Adolescents. Front Neurol. 2019;10:395. Epub 2019/05/21. doi: 10.3389/fneur.2019.00395 ; PubMed Central PMCID: PMC6492460. 31105634 PMC6492460

[46] Lawrence DW. Concomitant Persistent Symptoms Postconcussion and Infectious Mononucleosis: A Case Report. Curr Sports Med Rep. 2022;21(1):12–4. doi: 10.1249/JSR.0000000000000925 . 35018893

[47] Ledoux AA, Barrowman N, Bijelic V, Borghese MM, Davis A, Reid S, et al. Is early activity resumption after paediatric concussion safe and does it reduce symptom burden at 2 weeks post injury? The Pediatric Concussion Assessment of Rest and Exertion (PedCARE) multicentre randomised clinical trial. Br J Sports Med. 2021. Epub 20211126. doi: 10.1136/bjsports-2021-105030 . 34836880

