# Supplementary material for: Randomized controlled trial of early aerobic exercise following sport-related concussion: Progressive percentage of age-predicted maximal heart rate versus usual care
Source: PLoS One. 2022 Dec 22;17(12):e0276336. doi: 10.1371/journal.pone.0276336 (PMC9778585; doi:10.1371/journal.pone.0276336)
Supplement: S1 File — Full protocols for the Structured Aerobic Exercise and Usual Care Exercise groups, as well as expanded statistical analysis encompassing model form, prior selection, and data transformations. (DOCX) [file pone.0276336.s001.docx]

**Supplementary Methods**

**Structured Aerobic Exercise Protocol**

The first exercise session was performed at 60% and consisted of a 5-minute warm-up, 15-minutes of exercise at the prescribed intensity, and a 5-minute cool-down (25 minutes total). For each subsequent exercise session, the duration was increased to a total of 30 minutes (5-minute warm-up, 20-minute exercise, and 5-minute cool-down). Twenty minutes of exercise was chosen based on our prior feasibility study [1], as well as past studies that have evaluated aerobic exercise following concussion [2, 3]. The participants were required to successfully complete two exercise sessions at each respective intensity to progress to the next exercise intensity level. The exercise session was terminated and classified as a failed attempt if the participant’s symptoms increased by more than three points on the total symptom severity score from the symptom checklist of the SCAT5. If the participant failed an attempt, they were advised to reattempt the same exercise session the following day (i.e., intensity and duration). As a safety precaution, if the participant failed two consecutive exercise sessions, they were removed from the study and continued with usual care for the remainder of the study; their data was censored. Additionally, adverse events with any participants were required to be reported immediately to the clinical staff/physician and to the principal investigator (MGH).

**Usual Care Exercise Protocol**

*General Principles*

- Each stage of exercise should be performed without symptom production either during or after (within an hour or so) exercise.
- If a stage of exercise does produce symptoms, then the next session should the patient has to “go back” to the last stage that did not produce symptoms, and subsequently progressed with caution, after a day or two of asymptomatic exercise.

*By Whom*

- Ideally, such an exercise progression should be supervised and monitored by a qualified health professional.
- However, patients may “supervise” their own progression if needed, so long as they understand both the principles involved, and the specifics.

*How Often to Exercise:*

- Sessions in an exercise progression after concussion are typically once daily.
- In some cases (either severe injuries with prolonged symptoms, or mild injuries with

fleeting symptoms) the frequency of these sessions may be reduced (to several sessions per week) or increased (to twice daily) as may appropriate in the clinical judgement of a supervising health professional.

*How Quickly to Progress:*

- This is not a time-based process; rather, it is based on successfully reaching “milestones”. Each stage is commenced only after the last stage has been completed successfully.
- There is no “correct” rate of progression so long as the previous stage has been successfully passed.
  - In the mildest cases with quickest recovery, an athlete may potentially progress from one stage to another on the same day (if doing two sessions of exercise that day).
  - In severe or prolonged cases, it is generally prudent to perform exercise successfully (without symptoms or exacerbation) for at least several sessions before progressing to the next stage.

*Stages of Exercise Progression*

1. *Rest*
   Goal: Reduction of symptoms / attainment of asymptomatic state. Description:
   No physical activities that raise HR above resting rate.
   Should perform non-vigorous isometric stabilization exercises for neck.
2. *Re-Introduction of Stressor*
   Goal: Establish capacity for cardio-respiratory exertion without symptom production
   (or symptom exacerbation if performing progression despite persistent symptoms).
   Description:
   Stationary bicycle (no head movement) with progression of intensity or duration (not concurrently in one session).

a. 15 minutes @ 120 bpm
b. increase duration to 30 minutes @ 120 bpm
c. 30 minutes @ 140 bpm
d. 30 minutes @ 140 bpm with 1-minute maximal sprints every 5 minutes @ 5,10, 15, 20, 25 min)

1. *Low Level Stress*
   Goal: Establish capacity for at least 30 minutes per day of sport-specific movements that involve progressively greater acceleration of the head without symptom production (or exacerbation) Description:
   Sport specific movement that is most relevant to an patient’s function, with gradual progression of both intensity of exercise and magnitude of head acceleration, individualized as appropriate. At this stage there should be no “cognitive burden” where drills require them to make decisions or concentrate on a task other than basic movement. No resistance training.
2. *Intermediate Stress*
   Goal: Establish that at least 30 minutes per day of sport-specific training (stage 3) with additional cognitive and visual tracking burdens and body-weight resistance exercises does not produce or exacerbate symptoms.
   Description:
   Continue with the intensity and duration of sport-specific drills reached in stage 3, but now add an element of thinking or decision-making, such as ball or puck-handling, shooting, passing, positional play, execution of set plays, and so on. Also, add 15 minutes per day of body-weight floor exercises such as planks, push-ups, squats, lunges, sit-ups, box jumps, and so on – progress these from less intense (e.g. planks) to more intense (e.g. box jumps). Do not hold breath.
3. *Submaximal Stress*
   Goal: Establish freedom from symptoms in situations that simulate all aspects of normal training, except for the risk of collision.
   Description:
   Sport-specific drills should involve increased complexity simulating game situations. Increase duration toward normal daily training load for this sport. Resistance training may now use additional weights or machines as would be typical of training programs in your sport. Heavy resistance exercises may involve Valsalva manoeuvre (breath-holding during very heavy lifting, which increases intracranial pressure).
4. *Full Physical Activity/Sport*
   Goal: Unrestricted activity without symptom production. Description:
   Full participation in sports.

**Statistical Analysis**

The survival curve employed in the current study uses the exponential distribution, which contains a single parameter, rate (λ), which can also be expressed as the average displacement (λ^-1^) [4]. Displacement was measured as the time to asymptomatic status calculated separately in both groups, followed by the evaluation of a contrast (difference). However, as not all study participants were asymptomatic by day 28 – including participants who were removed from the SAEP group due to symptom exacerbation or who were lost to follow-up – the survival model had to account for censored events. Hence, a cumulative probability distribution was used to model the proportion of participants who became asymptomatic on or before any given day from day 1 to 28, while a complementary cumulative probability distribution (CCDF; one-minus the cumulative distribution) modelled the censored events by providing the probability of not being asymptomatic by the same number of days. Therefore, the model was of the form,

Days_i_|Asymptomatic status reached_i_ ~ Exponential(λ_i_)

Days_i_|Asymptomatic status NOT reached_i_ ~ Exponential-CCDF(λ_i_)

λ = 1/μ

log μ_i_ = α_GROUP[i]_

where the CCDF is the complementary cumulative distribution function, and μ is the average time to asymptomatic status. Weakly regularizing priors were employed with an *a priori* expected average time to asymptomatic status set at ~28 days, with room for substantial variance (~ 21 days). This was chosen based on prior studies conducted by our lab that have found similar recovery times and variability [5]. Hence, a gaussian prior with mean and standard deviation (SD) parameters of the form α ~ Normal(3.15, 0.6) was used; prior simulations yielded an average time to asymptomatic status of ~ 28 days, with a SD ~ 18 days (**S2 Fig**). Similarly, an exponential model was used to evaluate the expected time to medical clearance. This model used the same priors as the model evaluating time to asymptomatic status, however, censoring was performed on four participants who remained in the study but were missing medical clearance dates.

For cross-classified linear regression models comparing symptom severity, total symptoms, and percent of normal variables, contrasts between groups at each time point were created from the time and group intercepts. The Widely Available Information Criterion (WAIC) test was used to compare a non-pooled model to a hierarchical model that pooled the time intercept (weeks 1 – 4) with an adaptive gaussian prior; for all models, hierarchically modelling the time intercept offered no gain in model information compared to a non-hierarchical model, and as such the latter model was used. For symptom severity, values were square root transformed, with the priors set at α[time] ~ Normal(3, 1), α[group] ~ Normal(2, 1), and the standard deviation set at σ ~ Exponential(1). Prior simulations yielded no difference between groups (mean symptom score of ~ 27 in both groups), across all assessments (S3 Fig). Total symptom values were also square root transformed prior to evaluation, and priors were set at α[time] ~ Normal(1.25, 1), α[group] ~ Normal(1.25, 1), with the standard deviation set at σ ~ Exponential(1). Prior simulations for the total symptoms model also yielded no difference between groups at any assessment, with total symptom scores of ~ 14 in both groups (S3 Fig). Finally the percent of ‘normal’ variable was z-transformed prior to modelling, with priors α[time] ~ Normal(0, 0.5), α[group] ~ Normal(0,0.5), and the standard deviation set at σ ~ Exponential(1). Prior simulations yielded no difference between cohorts at any assessment, with a value of ~75% of ‘normal’ in both groups (S3 Fig).

All HMC simulations were run across four chains at 20000 iterations per chain. Convergence was measured using the Gelman-Rubin convergence test, with all chains converging to <1.01 [6]. (S4 Fig)

**References**

1. Micay R, Richards D, Hutchison MG. Feasibility of a postacute structured aerobic exercise intervention following sport concussion in symptomatic adolescents: a randomised controlled study. BMJ Open Sport Exerc Med. 2018;4(1):e000404. Epub 2018/07/19. doi: 10.1136/bmjsem-2018-000404. PubMed PMID: 30018795; PubMed Central PMCID: PMCPMC6045733.

2. Leddy JJ, Haider MN, Ellis MJ, Mannix R, Darling SR, Freitas MS, et al. Early Subthreshold Aerobic Exercise for Sport-Related Concussion: A Randomized Clinical Trial. JAMA Pediatr. 2019;173(4):319-25. doi: 10.1001/jamapediatrics.2018.4397. PubMed PMID: 30715132; PubMed Central PMCID: PMCPMC6450274.

3. Leddy JJ, Master CL, Mannix R, Wiebe DJ, Grady MF, Meehan WP, et al. Early targeted heart rate aerobic exercise versus placebo stretching for sport-related concussion in adolescents: a randomised controlled trial. The Lancet Child & Adolescent Health. 2021. doi: <https://doi.org/10.1016/S2352-4642(21)00267-4>.

4. McElreath R. Statistical Rethinking: A Bayesian Course with Examples in R and Stan: CRC Press; 2020.

5. Lawrence DW, Richards D, Comper P, Hutchison MG. Earlier time to aerobic exercise is associated with faster recovery following acute sport concussion. PLoS One. 2018;13(4):e0196062. Epub 2018/04/19. doi: 10.1371/journal.pone.0196062. PubMed PMID: 29668716; PubMed Central PMCID: PMCPMC5905975.

6. Vehtari A, Gelman A, Simpson D, Carpenter B, Bürkner P-C. Rank-Normalization, Folding, and Localization: An Improved Rhat for Assessing Convergence of MCMC. Bayesian Analysis. 2020. doi: 10.1214/20-ba1221.
