# Supplementary material for: Randomized controlled trial of early aerobic exercise following sport-related concussion: Progressive percentage of age-predicted maximal heart rate versus usual care
Source: PLoS One. 2022 Dec 22;17(12):e0276336. doi: 10.1371/journal.pone.0276336 (PMC9778585; doi:10.1371/journal.pone.0276336)
Supplement: S3 File — Study protocol. (DOCX) [file pone.0276336.s003.docx]

**Post-acute structured exercise following sport concussion**

Principal Investigator: Michael G. Hutchison, PhD RKin

Funded by: Canadian Institutes of Health Research (CIHR)

Draft or Version Number: v.1.1

27-December-2021

Table of Contents

[1.0 STATEMENT OF COMPLIANCE 4](#_Toc98429241)

[2.0 PROTOCOL SUMMARY 5](#_Toc98429242)

[3.0 SCHEMATIC OF STUDY DESIGN 6](#_Toc98429243)

[4.0 KEY ROLES 7](#_Toc98429244)

[5.0 BACKGROUND INFORMATION 8](#_Toc98429245)

[6.0 THE NEED FOR A TRIAL 8](#_Toc98429246)

[6.1 Pathophysiology following SRC 8](#_Toc98429247)

[6.2 Current concussion management 9](#_Toc98429248)

[6.3 Questioning rest after SRC 9](#_Toc98429249)

[6.4 Utility of Exercise in CNS Injuries 9](#_Toc98429250)

[6.5 Utility of exercise after concussion 9](#_Toc98429251)

[6.6 Knowledge Gaps 10](#_Toc98429252)

[7.0 POTENTIAL BENEFITS AND RISKS 10](#_Toc98429253)

[8.0 OBJECTIVES 11](#_Toc98429254)

[9.0 STUDY DESIGN 12](#_Toc98429255)

[9.1 Participants 12](#_Toc98429256)

[9.2 Intervention 13](#_Toc98429257)

[9.3 Inclusion 13](#_Toc98429258)

[9.4 Exclusion 13](#_Toc98429259)

[9.5 Structured Aerobic Exercise Protocol (SAEP) 13](#_Toc98429260)

[9.6 Measures 14](#_Toc98429261)

[9.6.1 Clinical Assessment: 14](#_Toc98429262)

[9.6.2 Physician and Research Assistant Blinding: 14](#_Toc98429263)

[9.6.3 Assessments at Day 7, 14, 21, 28, and 90: 14](#_Toc98429264)

[10.0 TRIAL MANAGEMENT 15](#_Toc98429265)

[11.0 STUDY ENROLMENT & WITHDRAWEL 15](#_Toc98429266)

[12.0 ADVERSE EVENT (AE) REPORTING 16](#_Toc98429267)

[13.0 SAMPLE SIZE 16](#_Toc98429268)

[14.0 DESCRIPTION OF STATISTICAL METHODS 16](#_Toc98429269)

[14.1 Analysis of Primary Endpoints 16](#_Toc98429270)

[14.2 Analysis of Secondary Endpoints 17](#_Toc98429271)

[15.0 INSTITUTIONAL REVIEW BOARD 17](#_Toc98429272)

[16.0 REFERENCES 18](#_Toc98429273)

[17.0 Appendix 23](#_Toc98429274)

# 1.0 STATEMENT OF COMPLIANCE

The Principal Investigator will assure that no deviation from, or changes to the protocol will take place without documented approval from The University of Toronto's Research Ethics Boards, except where necessary to eliminate an immediate hazard(s) to the trial participants.

I agree to ensure that all staff members involved in the conduct of this study are informed about their obligations in meeting the above commitments.

Principal Investigator: Michael Hutchison

Signed: _____________________________________

# 2.0 PROTOCOL SUMMARY

Title: Post-acute Structured Exercise Following Sport Concussion: a Randomized Controlled Study

Overview: This study will investigate the effect of structured, structured aerobic exercise protocol (SAEP) compared to usual care on clinical recovery from sport-related concussion (SRC) within the post-acute phase of injury. Participants will be randomized into one of two groups: (1) Supervised Exercise Group: participants will complete a total of eight exercise sessions over the course of 11 days, starting at Day 3 post-injury (two sessions (first and mid-point) will be done in the lab, and the remained will be home-based sessions); (2) Usual Care Group: individuals will undergo a period of physical rest and standard care. For the purposes of this study, "rest" will be defined as the avoidance of any activities beyond those of daily living, including participation in sport and physical activity.

Objectives: *Primary:* To investigate the utility of an SAEP initiated in the sub-acute phase after SRC in reducing post-concussion symptoms and the time to medical clearance, compared to usual care. *Secondary:* To determine the effect of an SAEP in concussed subjects on ANS function, blood biomarkers, and cognition

Outcome Measures: *Primary*: post-concussion symptom scores and days to medical clearance; *Secondary:* a) cardiovascular responses [heart rate variability (HRV) and blood pressure variability (BPV)], b) cognitive performance, and c) neuroinjury/neuroreparative blood biomarkers (BDNF, glial fibrillary acidic protein [GFAP], peroxiredoxin [PRDX]-6, monocyte chemoattractant protein [MCP]-1), and catecholamines (norepinephrine [NE] and epinephrine [Epi]).

Endpoints: *Primary:* Days to asymptomatic status and days to medical clearance *Secondary:* Cardiovascular, cognitive, and neuroinjury markers at Day 90 post-injury.

Population:

Age: 13-25 years, Sex: All

Inclusion Criteria:

Diagnosed with SRC by a physician at the David L. MacIntosh Sport Medicine Clinic

Able to speak and understand English

Exclusion Criteria:

Have had a previous concussion within two weeks of the presenting SRC

Have any co-morbid injuries (i.e. musculoskeletal/soft-tissue injuries, vestibular disorders)

Have a pre-existing heart condition

Have any uncontrolled seizure disorders or a history of medical or neurological conditions that affects cognitive functioning

Number of Sites: 1

Study Duration: 36 months

Participant Duration: 90 days

# 3.0 SCHEMATIC OF STUDY DESIGN

# 4.0 KEY ROLES

**Prof. Michael Hutchison** will be responsible for project management and progress. Dr. Hutchison has experience in all aspects of the study, with publications spanning clinical recovery ^1^, autonomic dysfunction^2,3^, cognitive testing^4-6^, and blood biomarkers^7-10^. As Medical Directors, **Dr. Doug Richards and David Lawrence** ensure physicians and staff are familiar with the study protocol regarding recruitment, scheduling clinical follow-ups, and physician blinding. **Dr. Scott Thomas** has experience with exercise intervention protocols in clinical populations ranging from stroke to heart disease, with expertise in HRV and BPV, clinical trials and data analysis. **Dr. Shawn Rhind** will oversee and provide the resources and expertise for analyses and interpretation of blood biomarkers. **Dr. Alex Di Battista** will coordinate the blood collection and analysis of blood biomarker data in association with Dr. Rhind. Research assistants **Ms. Kyla Pyndiura** and **Ms. Danielle Corrallo** are responsible for recruitment, data collection, and data management. **Mr. Patrik des los Reyes** is the clinical support staff member that will provide research assistants of potential patients interested in the participating in the study.

# 5.0 BACKGROUND INFORMATION

Concussions are a significant health concern occurring frequently in athletic setting^11,12^. The literature commonly cites spontaneous recovery within 7-10 days, however, this evidence has been historically limited to sport-related concussions (SRC) among US collegiate and professional athletes^13^. A recent meta-analysis and data from Canadian pediatric emergency departments document significantly longer recovery times, particularly for younger individuals^14,15^. Current medical guidelines advise complete physical and cognitive rest acutely after SRC, followed by gradual re-introduction of activity in a standardized manner^16^. Notably, the most recent Concussion in Sport Consensus Statement highlighted that the exact amount and duration of rest is not yet well defined in the literature and requires further study^16^.

The current standard care after concussion is largely predicated upon experimental animal studies, which have shown a decrease in energy availability in the brain resulting from neurometabolic disruption^17^. From this, it has been speculated that any activity after injury may exacerbate this hypermetabolic state, increase symptoms, and delay recovery^18,19^. However, recent evidence suggests there is no added benefit of rest on symptom recovery after concussion beyond 3 days^18^, and inactivity may contribute to the development of maladies often concomitantly present in concussion sufferers, such as mental health conditions, chronic fatigue, and pain disorders^18^.

A growing body of research has demonstrated that early-onset, graded aerobic exercise (AE) interventions following central nervous system (CNS) injuries such as stroke, whiplash, and low-back pain, can accelerate recovery and improve functional outcomes compared to rest alone^18,20-24^. While the underlying therapeutic mechanisms are unclear, it is thought that exercise may expedite parenchymal tissue regeneration and restore dysregulated autonomic system (ANS) function^3,21,25-31^. In traumatic brain injury (TBI), early AE intervention in animal models has shown improved cognitive recovery and alleviation of symptoms, alongside numerous biological alterations such as increased neurogenesis, decreased apoptosis in the hippocampal regions, enhanced survival of purkinje fibres, and suppression of reactive gliosis^29,32-36^.

While preliminary human investigations using AE interventions after concussion have shown promise, these studies are either retrospectively designed^37^, or have focused on individuals with persistent symptoms (> 3 weeks)^38-40^. Given the promising evidence of the therapeutic potential of AE interventions, and the large percentage of adolescents and young adults at risk for concussion^16,41,42^, the purpose of this study is to examine the effect of a standardized, subacute AE intervention on recovery after SRC compared to usual care. This study will provide meaningful information regarding the utility and optimal implementation of AE intervention after concussion. Findings from these works may inform future usual care procedures post-injury, potentially providing the first known treatment to improve recovery after concussion. Furthermore, as AE is easily implementable and not resource intensive, its use in the treatment of concussion could have widespread impact.

# 6.0 THE NEED FOR A TRIAL

## 6.1 Pathophysiology following SRC

The underlying mechanisms mediating recovery following SRC are not well understood. However, evidence suggests the initial trauma leads to neurometabolic disruption, neuroinflammation, and autonomic nervous system (ANS) dysregulation ^17,43-45^.

After injury, trauma to neuronal tissue, particularly as a result of shearing forces, leads to a cascade of events including microporation of lipid cellular membranes, glutamate release from damaged cells, depletion of intracellular energy, and calcium mediated parenchymal tissue damage^17^. Additionally, the neuroinflammatory response is activated, which can paradoxically lead to the beneficial removal of extracellular glutamate, neurogenesis, angiogenesis and axonal remodeling, but may also facilitate detrimental processes such as the overproduction of cytokines, chemokines, reactive oxygen species (ROS), and the isolation of injured areas of the brain from healthy tissue^46,47^. Consistent with this, employing advanced neuroimaging and peripheral blood biomarker analysis, our group and others have identified evidence of neurometabolic disruption, CNS injury, and inflammation across the spectrum of human TBI^7-10,48-53^. Furthermore, concussion may also cause dysfunction to the central autonomic system (ANS) due to sheer forces form the insult, resulting in subtle structural alterations in brainstem regions responsible for autonomic modulation^19^. Indeed, heart rate variability (HRV) impairments have been observed in mild traumatic brain injury (mTBI) patients ^54^, and our group recently identified a decrease in HRV in athletes post-SRC^3,7-10,48^. Notably, we have also found numerous pathological relationships between sympathetic hyperactivity and secondary injury processes in TBI patients ^7,9^.

## 6.2 Current concussion management

Concussion is a disturbance in brain function caused by direct or indirect force to the head. Therefore, the diagnosis of concussion requires determination of a temporal relationship between an appropriate mechanism of injury and signs and/or symptoms. Most recent management guidelines for mTBI still universally recommend an initial period of rest^55,56^; this is reflected in the consensus statement derived from the 5^th^ international conference on Concussion in 2016, where it is recommended that following a period of rest after injury, patients may be encouraged to become gradually and progressively more active while staying below their cognitive and physical symptom-exacerbation thresholds^16^. Unfortunately, the appropriate intensity, frequency, and duration of activity is unclear. In addition, given that too much activity has been associated with worse outcomes^57^, careful investigation is needed in order to inform future evidence-based guidelines.

## 6.3 Questioning rest after SRC

Observational studies have consistently documented the negative consequences of inactivity in various conditions^18^. Sedentary behavior following injury/illness is the most consistent risk factor for chronic disability^58^, and prescribed rest may begin to adversely affect the cardiopulmonary and musculoskeletal systems in healthy people within just three days^59^. Inactivity may also exacerbate and/or prolong recovery in commonly identified mTBI comorbidies such as vestibular disorders, mental health disorders, chronic fatigue syndrome, and pain disorders^18^. Athletes in particular are vulnerable to the deleterious effects of prolonged rest with respect to physical deconditioning, as well as secondary symptoms arising from fatigue and reactive depression^31^.

## 6.4 Utility of Exercise in CNS Injuries

The positive effects of structured AE on recovery exists for a number of CNS injuries^18^. Exercise has been shown to influence multiple neurotransmitter systems and promote neuroplasticity, neurogenesis, and angiogenesis following a traumatic event^20,22^. Specifically, limiting bed rest and initiating AE has been associated with positive outcome for stroke^60,61^, whiplash^62,63^, and low-back pain^64,65^ patients.

The evidence supporting AE intervention after TBI is promising, but predominantly limited to animal experimentation. The initiation of exercise training shortly after TBI in rats may be an important facilitator of recovery from cerebral dysfunction, due to neurogenesis and the proliferation of neuronal stem cells surrounding the injured site^29,66^. In addition, voluntary AE initiated immediately following injury has been shown to significantly inhibit apoptosis of neurons and decrease hippocampal DNA fragmentation in injured areas of the brain, leading to improvements in cognitive function and memory^35,67^. Lastly, AE following TBI has also been associated with enhanced survival of Purkinje fibres and the suppression of reactive astrocyte formation^36^.

## 6.5 Utility of exercise after concussion

AE has benefited adults and children who are slow to recover after concussion^40,68^. The scientific basis for recommending AE in the treatment of concussion includes its ability to improve cortical connectivity and activation^69^, facilitate neurogenesis, and regulate cerebral blood flow (CBF) and reactivity.

Exercise is associated with greater levels of brain-derived neurotrophic factor (BDNF), which is involved in neuron repair, increasing hippocampal volume, and improving spatial memory^70^. Moreover, as prolonged rest after TBI may lead to physical deconditioning of the cardiovascular system and a resultant impairment of CBF control, exercise training and physical fitness can improve CBF control and cerebral vasoreactivity^71^.

Functional magnetic resonance imaging (fMRI) studies show an excess of metabolic activity during simple cognitive tasks post-injury^72,73^, suggesting decreased efficiency in the concussed brain. AE treatment has been shown to restore fMRI brain activation patterns to normal compared to a sham (stretching) program, and is associated with persistent symptom resolution, including fatigue^40^. Similarly, Gagnon and colleagues found that the implementation of an active rehabilitation intervention was associated with significantly decreased post-concussion symptoms, as well as decreased fatigue and improved mood in a group of adolescents who were still symptomatic at four weeks post-SRC.^74^

Despite the promise of the benefit of exercise in those with persistent symptoms, there is a void of prospective studies investigating the efficacy of graded AE rehabilitation strategies in concussion patients at the sub-acute phase of recovery. In view of this, a retrospective study by Majerske and colleagues found that athletes engaging in a medium level of physical and cognitive activity post-injury had fewer symptoms and performed better on neurocognitive testing compared to those with no activity and the highest levels of activity^37^. These findings further support the potential benefit of exercise post-concussion, and warrant future prospective investigations.

## 6.6 Knowledge Gaps

As adolescents and young adults represent the overwhelming majority of concussion patients^75,76^, the development of a safe, effective, and evidence-based rehabilitation strategy is imperative for this population. Yet, despite emerging evidence demonstrating the efficacy of exercise interventions in the treatment of various CNS injuries (i.e. stroke, whiplash, low back pain, dementia), very few studies have examined the clinical utility of alternative rehabilitation strategies beyond rest. While AE interventions have been evaluated in patients with prolonged symptomology, investigations on the effect of structured exercise early post-concussion have been limited to animal models of injury, or assessed in humans using a retrospective study design. Furthermore, these studies rely mainly on subjective reports of concussion-associated symptoms to indicate impairment, and generally do not involve objective biological markers of underlying pathological processes.

# 7.0 POTENTIAL BENEFITS AND RISKS

Physical Risks During the study, participants in the Structured Aerobic Exercise Protocol (SAEP) group may experience an increase in their symptoms due to physical exertion. Should symptoms increase during a session, the session will be terminated immediately. If a participant in the exercise group experiences an increase in their symptoms over the course of two consecutive sessions, then their participation in the SAEP intervention will be terminated. While these participants will be removed from the larger group analysis, careful evaluation of this potential subset will be conducted. In addition, these participants will remain in the study and they will complete assessments on Days 7, 14, 21, 28, and 90 post-injury. In order to ensure the safety of all participants, there will be an on-call physician at all times available at the David L. Macintosh Sports Medicine Clinic, which is located directly next to the lab space in which the exercise sessions will take place.

Participants in the SAEP may also experience symptom exacerbation later in the day following the exercise intervention, even after they have left the testing facility. In order to minimize this risk, participants will be kept at the facility for an additional 10 minutes following the completion of the entire exercise session (including the 5 minute cool-down period) in order for the supervising researcher to monitor their symptoms and ensure that they their HR and BP have returned to pre-exercise levels. Additionally, participants in this group will be provided with the contact information for the David L. Macintosh Clinic, where a sports-medicine physician will be available on-call at all times should the participant require urgent care after they have left the facility. Finally, there may be minor and temporary discomfort associated with the blood-drawing procedure. This may include soreness or bruising of the skin in area of the blood-draw. This should resolve within a few days after the blood draw. Risks of experiencing infection or irritation of the skin surrounding the site of the blood acquisition will be minimal due to the training of the phlebotomist on site. If a sample cannot be acquired due to difficulties in locating a vein, or if a vein collapses, we will ask the participant if they are comfortable with one additional attempt on the opposite arm. If they are not comfortable with this, or if we are unable to acquire a sample on the other arm, we will terminate the blood acquisition portion of the assessment for the participant. Psychological/Emotional Risks Participants may become upset or frustrated if they lack progress in their perceived recovery time. In order to mitigate the potential mental health-associated risks that may arise due to participation in this study, participants’ will be given priority scheduling to be seen by a physician at the Macintosh Clinic, who will monitor them for mental health-related symptoms and refer them for psychiatric care if necessary. In addition to including the contact information for the Macintosh Clinic, the informed consent documents have been updated to provide participants with the contact information for local mental health treatment centres in the area that they may access should they require additional psychiatric resources.

Participants in both groups (Usual Care, and SAEP) will benefit from their participation in this study with respect to enhanced education on concussion and the various assessment measures associated with it (i.e. HRV, BPV, symptoms, and peripheral serum sampling). Participants will also benefit from this study as it will give them the opportunity to be seen by a sport-medicine physician every week for four weeks, which is far more medical attention than a normal concussion patient would receive. These patients would also be offered priority scheduling at the Macintosh Clinic. Furthermore, participants who follow the standardized exercise protocol who then may experience a faster recovery time than those managed with the current RTP management protocol, based on the evidence brought forth by previous literature in the field of neurorehabilitation and exercise. Moreover, participants in the exercise group will be given access to exercise equipment and supervision they would not otherwise be able to make use of. We anticipate that this study will facilitate significant progress to the sports medicine community. As a result of this study, our research team will be able to empirically compare both novel and current RTP management protocols. Given the lack of clinically validated rehabilitation strategies that currently exist in the management of SRC, this study has the potential to provide evidence of the clinical utility of a standardized, structured AE rehabilitation program for patients in the post-acute phase of recovery from SRC.

# 8.0 OBJECTIVES

**Specific Aim 1:** To investigate the utility of an Structured Aerobic Exercise Protocol (SAEP) initiated in the sub-acute phase after SRC in reducing post-concussion symptoms and the time to medical clearance, compared to usual care.

***Hypothesis 1 – Clinical recovery:***

Individuals in the SAEP will have lower symptom scores and faster return to asymptomatic status, leading to shorter clinical recovery times compared to those in usual care group.

**Specific Aim 2:** To determine the effect of an SAEP in concussed subjects on ANS function, blood biomarkers, and cognition.

***Hypothesis 2a*** – Cardiovascular responses:

Compared to the SAEP, the usual of care group will display lower HRV and BPV at days 14, 21, and 28 post-SRC.

***Hypothesis 2b:*** *- Peripheral blood biomarkers:*

As compared to the usual of care group, the SAEP group will display higher peripheral concentrations of neuroreparative indices BDNF and PRDX-6, lower concentrations of the CNS-injury analogue GFAP, lower concentrations of the inflammatory marker MCP-1, and lower resting peripheral catecholamine levels.

***Hypothesis 2c –Cognition:***

Compared to the usual care group, the SAEP group will display significantly improved cognitive performance at days 14, 21, and 28 post-SRC.

# 9.0 STUDY DESIGN

**Design:** A single blinded, randomized, controlled trial with 7-, 14-, 21-, 28-, and 90-day follow-up visits.

**Setting:** David L. MacIntosh Sport Medicine Clinic at the University of Toronto.

**Main Outcome Measures**: *Primary outcome measures*: post-concussion symptom scores and days to medical clearance; *Secondary outcome measures:* a) cardiovascular responses [heart rate variability (HRV) and blood pressure variability (BPV)], b) cognitive performance, and c) neuroinjury/neuroreparative blood biomarkers (BDNF, glial fibrillary acidic protein [GFAP], peroxiredoxin [PRDX]-6, monocyte chemoattractant protein [MCP]-1), and catecholamines (norepinephrine [NE] and epinephrine [Epi]).

**Intervention**: Structured Aerobic Exercise Protocol or Usual Care.

## 9.1 Participants

Participants will be recruited from the David L. Macintosh Sport Medicine Clinic (University of Toronto). The Macintosh Clinic is an interdisciplinary sport medicine clinic with a Concussion Program. The clinic is known for their acute management of concussions and is the primary clinical site for University of Toronto students who sustain physical activity related injuries. The clinic also has a number of existing relationships with local district schoolboards and sports organizations. These organizations are not founded on research participation, and do not require physician referral for concussion. The most recent estimate of new concussion cases in the clinic per year is approximately 300-350. Based on our previous research conducted at the MacIntosh Clinic^1,2^, as well as recent Canadian emergency department statistics^14^, we expect female participants to account for 40% of our study sample. Potential study participants will be examined and diagnosed with SRC within 72 hours of injury by one of the 3 physicians working at the clinic who specialize in concussion management. If the patient is diagnosed with a concussion, informed consent and study enrolment will occur after the first office visit. At Day 3 post-concussion, study eligibility will be confirmed by a Post-Concussion Symptom Scale (PCSS) evaluation used to determine their symptom status. The decision to wait three days post-injury to randomize participants and initiate the intervention is aligned with an approach based on the suggestion that rest beyond three days following mTBI is provides no further benefit to recovery^16^. At that time, symptomatic patients (defined as having a PCSS score equal to or greater than five on the SCAT5) will be randomly divided into one of two groups by a computer generator; this task will be overseen by a member of the research team not involved in the consent process. Our operational definition of “symptomatic” status (PCSS score 5 or greater) is based on prior literature, including a systematic review by Alla and colleagues, which suggests low PCSS scores are representative of the general population’s symptom presentation in a non-concussed state^77^**.**

## 9.2 Intervention

**(1) Usual care (UCEP) group:** Participants allocated to the UCEP group were advised to follow the instructions, prescriptions, and recommendations given to them by the sport medicine physician. Aligned with consensus guidelines of a brief period of physical and cognitive rest, the physician advised participants to increase their activity levels gradually with minimal head movement (predominantly involving a stationary bike) and progressively increase levels of exertion while remaining under the threshold of symptom exacerbation.^78^ Subsequently, exercise included a progression of head movements, visual and cognitive burdens, sport-specific activities, and heavy resistance, in that order, all below the symptom exacerbation threshold. For more details on the UCEP, please see the **Appendix**. Any diagnosed concomitant neck injuries underwent concurrent rehabilitation. Completion of asymptomatic low-risk simulations of target activities was required prior to medical clearance to return to those activities.

**(2) Structured Aerobic Exercise Protocol (SAEP) group:** These individuals will begin to exercise at Day 3 post-injury. Participants will be asked to complete a total of eight exercise sessions over the course of 11 days, with one day of rest after two consecutive sessions. The participants will have their symptoms recorded before and after exercise, and monitored throughout each session. Symptoms will only be recorded during exercise if exacerbated, in which case the session will be terminated. If this occurs, patient will resume the same stage in the protocol at the subsequent session. If the patient’s symptoms become exacerbated for two consecutive sessions, they will be removed from the study and continue with clinic for patient follow-up. Should the participant achieve asymptomatic status at any point, they will continue with the exercise protocol until completion, while simultaneously being guided through the RTP guidelines.

## 9.3 Inclusion

(1) Concussion diagnosis will be based on the following criteria: a) observed or reported acceleration/deceleration of the head; b) any observed alteration in mental status; and/or c) observed signs such as confusion, vacant stare, poor coordination, difficulty concentrating, poor balance; and/or d) any self-reported symptoms such as headache, loss of consciousness, nausea, balance problems or difficulty reading or concentrating; (2) Minimum grade 9 educational level; (3) Able to speak and understand English.

## 9.4 Exclusion

(1) Previous concussion within two weeks of the presenting SRC; (2) Presence of any co-morbid injuries (i.e. musculoskeletal/soft-tissue injuries, vestibular disorders); (3) Pre-existing heart condition or any uncontrolled seizure disorders; (4) History of medical or neurological conditions that affect cognitive functioning.

## 9.5 Structured Aerobic Exercise Protocol (SAEP)

The proposed exercise protocol will consist of 8 sessions, proceeding in a stepwise fashion with respect to duration and intensity over the course of 11 days, with two consecutive sessions proceeded by one day of rest. A fixed wattage stationary bike (Velotron Dyndafit Pro, manufactured by Racermate Inc., Seattle, WA, USA) will be used for all exercise sessions. The use of a stationary bike will ensure head and neck stability, avoiding any unwanted accelerations of the head (cf. treadmill). Finally, the bike will be connected to an interactive heart rate monitor (Polar®, Kempele, Finland), allowing the research team to control the wattage of the bike for each individual subject.

The first session of the AE protocol will take place on the Day 3 post-injury, and will comprise 15 minutes of steady-state exercise at 60% of the participant’s age-predicted maximal HR (apHR), plus a warm-up and cool-down period (25 minutes total). The participant’s symptoms will be monitored before, during, and after exercise. If the participant’s symptoms are not exacerbated, they will proceed to the next exercise level, consisting of 20 minutes of steady-state exercise, at 60% of apHR, plus warm-up and cool-down (30 minutes total). Please see **Table 1.** below for a detailed summary of the exercise protocol. Participants’ HR and BP will be recorded before and after each exercise session. Symptoms will also be recorded before and after each exercise session using the PCSS, and a trained kinesiologist will monitor symptoms throughout the exercise session. A prepared script to monitor symptoms has been developed to ensure standardization. If participants in the exercise group achieve asymptomatic status at the two weeks post-injury, they will continue to progress through the AE intervention until they complete all eight sessions, and will also begin progressing through the existing clinical RTP guidelines.

In an effort to improve our ability to evaluate the efficacy of the intervention, all participants will be given an activity tracker. The benefit of providing an activity tracker is the ability to monitor and quantify activities with daily living (ADL) beyond the study protocol (Supervised Exercise Group or Usual Care Group). Participants will be provided activity tracker (Fitbit Alta HR) at the time of consent to participate in the study and returned to the researcher at Day 28 assessment. Data to be collected by Fitbit Alta HR include steps (daily) and heart rate (daily per beats per min); this data will be used as potential co-variates when evaluating the efficacy of the intervention.

**Table 1.** AE exercise protocol

|  |  |  | **AE Protocol** | | | | | | | | |
| --- | --- | --- | --- | --- | --- | --- | --- | --- | --- | --- | --- |
| Day | 3 | 4 | 5 | 6 | 7 | 8 | 9 | 10 | 11 | 12 | 13 |
| Session | 1 | 2 | Rest | 3 | 4 | Rest | 5 | 6 | Rest | 7 | 8 |
| Duration (min) | 15 | 20 |  | 20 | 20 |  | 20 | 20 |  | 20 | 20 |
| Intensity (% apHR) | 60 | 60 |  | 65 | 65 |  | 70 | 70 |  | 75 | 75 |

## Measures

9.6.1 Clinical Assessment: At the first medical visit post-injury, participants will be evaluated by one of three treating physicians at the MacIntosh Clinic. The clinical assessment includes Sport Concussion Assessment Tool-5 (SCAT5), which includes information about the mechanism of injury, medical history, symptoms (PCSS), cognitive abilities, physical examination, and additional injuries/co-morbidities. Clinical management and corresponding follow-up evaluations will be at the discretion of the caring physician. Date of medical clearance will be obtained from medical records.

9.6.2 Physician and Research Assistant Blinding: To minimize bias, physicians will be blinded to the randomization of participants to either SAEP or usual care groups. Furthermore, all participants will be specifically instructed not to inform their treating physician of their group assignment at any point throughout the duration of the study. In addition, the randomization procedure will be overseen by a member of the research team who is not involved with the consent process.

### 9.6.3 Assessments at Day 7, 14, 21, 28, and 90:

***HRV Assessment:*** Resting HRV will be based on variability in participants’ R-R intervals using the Polar heart rate V800 sports watch and corresponding chest strap heart monitor (Polar ®, QC, Canada). The research team will be able to extrapolate the ratio of low frequency (i.e. sympathetic activity) to high frequency (i.e. vagal activity) signals that appear on a beat-to-beat basis, per subject. Participants will be asked to lie down in the supine position to allow for set up and accommodation. HRV will be assessed for 5 minutes in the supine position, then, after a 1-minute accommodation period, a final 5 minute HRV assessment in the upright-seated position will be performed.

***BPV Assessment****:* BPV will be assessed non-invasively on a beat-to-beat basis using the Finapres NOVA figure cuff device (Finapres Medical Systems, Amsterdam, Netherlands) employing a similar protocol to the assessment of HRV; participants will undergo a 5 minutes accommodation period, at which point their BPV will be assessed for 5 minutes in the supine position. Following this, participants will be asked to assume an upright-seated position, followed by a one-minute accommodation period and an additional five-minute assessment period in the upright-seated posture. Time and frequency domain measures of BPV will be recorded and digitally uploaded for each posture (not including the accommodation periods), as well as throughout the transition from one posture to the next, in order to determine the balance of sympathetic versus parasympathetic influence on the ANS.

***Blood Samples:*** Peripheral blood samples (approximately 20 mL) will be drawn from participants by a trained phlebotomist. Blood will be drawn on days 7, 14, 21, 28, 90 and 180 days post-injury in both groups, but before the onset of exercise in the SAEP intervention group. Specimens will be immediately centrifuged to isolate the plasma/serum, and aliquots will be stored at -70^ο^C until subsequent analysis. High sensitivity multiplexed immunoassay will be employed to quantitate inflammatory cytokines and chemokines, and CNS-injury specific biomarkers. In addition, radioimmunoassay will be employed to evaluate numerous neuroendocrine hormones.

***Cognitive Function:*** Using an iPad-based assessment tool (C3 Logix, Cleveland, OH, USA), participants will complete a series of tests designed to measure reaction time, information processing speed, visual acuity, and postural stability. This will take approximately 10-15 minutes to complete. The C3 Logix iPad system consists of seven modules for evaluation and takes approximately 17 min to perform. Four of the modules are based on long-standing traditional tests that have been translated to electronic form (i.e., Simple and Choice Reaction Test, Trails Test A & B, and symbol digit modalities test). C3 Logix also includes the most recent version of the Standardized Assessment of Concussion, which has been developed and recommended by the Concussion in Sport Group, and disseminated after the 5th International Conference on Concussion in Sport^16^.

# 10.0 TRIAL MANAGEMENT

We recognize participants in the SAEP group may experience an increase in their symptoms due to physical exertion. Despite the current usual care recommendation of rest until symptoms resolve, there is no evidence in humans suggesting exercise after 72 hours of rest is harmful. Nonetheless, symptoms will be monitored throughout the exercise session by a trained kinesiologist using a prepared script to allow the supervising researcher to determine symptom exacerbation (change of >3 points on symptom checklist, adopted by previous AE studies for individuals with PCS)^39^. Should symptoms significantly increase during an AE session, it will be terminated immediately. Furthermore, if a participant in the AE group experiences a significant increase in symptoms over the course of two consecutive sessions, their participation will be terminated. To further ensure participant safety, the AE sessions will take place in a training facility adjacent to the MacIntosh Clinic; immediate access to a physician will be available if required.

# 11.0 STUDY ENROLMENT & WITHDRAWEL

Potential participants will be informed of the study by a member of the research team. During the initial phase of the appointment, when the potential participants is completing the initial concussion forms, a member of the research team will assist them and answer any questions they might have. At this time, the member of the research team will explain that they may be eligible for the study. If a member of the research team is unavailable, potential participants will be informed of the study by their diagnosing physician at their initial medical assessment post-injury, and will be given an information flyer and confirmation that they can be contacted by phone by a member of the research team.

To ensure that all potential participants and their parents/guardians are fully aware of what they are consenting to, a member of the research team will brief them in person about the study’s purpose, methods, and possible risks prior to having them sign the informed consent documents. At this point, potential participants and their parents/guardians will have the opportunity to ask any questions that they may have with respect to the study before consenting to participate. The member of the research team will take this opportunity to remind the potential participants and their parents/guardians that their choice to participate is entirely voluntary, and that they have the option to withdraw themselves from the study at any point without any negative repercussions. The primary informed consent document for this study clearly outlines the purpose, procedures and potential risks of participation in this study. The parents/guardians of potential participants who are under the age of 16 will be required to sign this form on their child’s behalf, in addition to signing a parental consent form. Additionally, children under the age of 16 will be required to sign a child assent form. This will ensure that all aspects of the study are clearly articulated to both the potential participants and their parents/guardians, and that everyone involved is fully aware of what they are consenting to.

In the event of a participant’s withdrawal from the study, their data will be safely discarded with no consequences to the participant. The patient will continue to be able to have access to customary care at the David L. MacIntosh Sport Medicine Clinic. Participants are permitted to withdraw from the study at any point during data collection

# 12.0 ADVERSE EVENT (AE) REPORTING

An AE may jeopardize the patient or participant and may require medical or surgical intervention. Examples of such medical events include allergic bronchospasm requiring intensive treatment in an emergency room or at home or convulsions that do not result in inpatient hospitalization. All AEs will be assessed by a MacIntosh Clinic physician using a protocol defined grading system.

**Mild** – Events require minimal or no treatment and do not interfere with the participant’s daily activities.

**Moderate** – Events result in a low level of inconvenience or concern with the therapeutic measures. Moderate events may cause some interference with functioning.

**Severe** – Events interrupt a participant’s usual daily activity and may require systemic drug therapy or other treatment. Severe events are usually potentially life-threatening or incapacitating.

# 13.0 SAMPLE SIZE

Sample size was determined from a power analysis incorporating published data by our group on days to medical clearance in high school patients evaluated at the MacIntosh Clinic (mean time to clearance = 43 days, SD = 20 days).^79^. Hence, to reduce time to medical clearance to 36 days (delta = 7), at a significance level of 0.05 and a at a moderate effect level (0.7), would require 53 participants per group.

# 14.0 DESCRIPTION OF STATISTICAL METHODS

## 14.1 Analysis of Primary Endpoints

We will statistically evaluate time to medical clearance between the standard of care and AE intervention groups by Cox regression (or proportional hazards regression). This approach allows one to control for the effect of several variables (i.e., sex/gender, age, initial PCSS), while evaluating a single outcome measure. Furthermore, we have the ability account for censor subjects who are removed from the study due to exercise-induced symptom exacerbation or attrition.

## 14.2 Analysis of Secondary Endpoints

Furthermore, A mixed model ANOVA will be used to evaluate possible differences in symptoms, HRV, BPV, cognitive performance and blood biomarkers between the standard of care group and AE intervention group (between group analysis) across 6 time points; 7, 14, 21, 28, and 90 days post-SRC (within group analysis). If the data fails the required normality assumption, the appropriate transformation will be performed on all data. We expect approximately 40% females in our study sample, allowing for differential responses between males and females. We will perform separate Kaplan Meier analyses in males and females on time to medical clearance between the standard of care and AE intervention groups. Likewise, we will perform separate mixed model ANOVA analyses in males and females to evaluate differences between AE intervention and standard care groups for all aforementioned secondary measures.

Regarding blood biomarker analysis, each individual biomarker will only be included for statistical quantitation if they fall within the lower and upper limits of quantitation (LLOQ and ULOQ, respectively) as delineated by the manufacturer. All samples will be run in duplicate, and excluded if the coefficient of variance (CV) between duplicate samples is > 25%. Furthermore, mean statistical analysis will only be performed on biomarkers that have quantifiable samples in > 50%, in all groups in question. A quantifiable sample is 1) within the manufacturers quantitative range, and 2) displays a CV between duplicates of < 25%. In instances where one or both groups contain <50% quantifiable samples, differences between these groups will be evaluated by χ^2^.

# 15.0 INSTITUTIONAL REVIEW BOARD

We have institutional ethics approval from the University of Toronto (reference #33459) and a registered trial with clinictrials.gov (NCT02969824).

# REFERENCES

1. Hutchison M, Mainwaring LM, Comper P, Richards DW, Bisschop SM. Differential emotional responses of varsity athletes to concussion and musculoskeletal injuries. *Clin J Sport Med*. Jan 2009;19(1):13-9. doi:10.1097/JSM.0b013e318190ba06

2. Senthinathan A, Mainwaring LM, Hutchison M. Heart Rate Variability of Athletes Across Concussion Recovery Milestones: A Preliminary Study. *Clin J Sport Med*. Jul 1 2016;doi:10.1097/JSM.0000000000000337

3. Hutchison MG, Mainwaring L, Senthinathan A, Churchill N, Thomas S, Richards D. Psychological and Physiological Markers of Stress in Concussed Athletes Across Recovery Milestones. *J Head Trauma Rehabil*. Sep 6 2016;doi:10.1097/HTR.0000000000000252

4. Bruce J, Echemendia R, Tangeman L, et al. Two baselines are better than one: Improving the reliability of computerized testing in sports neuropsychology. *Appl Neuropsychol Adult*. Sep-Oct 2016;23(5):336-42. doi:10.1080/23279095.2015.1064002

5. Echemendia RJ, Bruce JM, Meeuwisse W, Comper P, Aubry M, Hutchison M. Long-term reliability of ImPACT in professional ice hockey. *Clin Neuropsychol*. Feb 2016;30(2):328-37. doi:10.1080/13854046.2016.1158320

6. Hutchison M, Comper P, Mainwaring L, Richards D. The influence of musculoskeletal injury on cognition: implications for concussion research. *Am J Sports Med*. Nov 2011;39(11):2331-7. doi:10.1177/0363546511413375

7. Di Battista AP, Rizoli SB, Lejnieks B, et al. Sympathoadrenal Activation is Associated with Acute Traumatic Coagulopathy and Endotheliopathy in Isolated Brain Injury. *Shock*. Sep 2016;46(3 Suppl 1):96-103. doi:10.1097/SHK.0000000000000642

8. Di Battista AP, Rhind SG, Richards D, Churchill N, Baker AJ, Hutchison MG. Altered Blood Biomarker Profiles in Athletes with a History of Repetitive Head Impacts. *PLoS One*. 2016;11(7):e0159929. doi:10.1371/journal.pone.0159929

9. Di Battista AP, Rhind SG, Hutchison MG, et al. Inflammatory cytokine and chemokine profiles are associated with patient outcome and the hyperadrenergic state following acute brain injury. *J Neuroinflammation*. 2016;13:40. doi:10.1186/s12974-016-0500-3

10. Di Battista AP, Buonora JE, Rhind SG, et al. Blood Biomarkers in Moderate-To-Severe Traumatic Brain Injury: Potential Utility of a Multi-Marker Approach in Characterizing Outcome. *Front Neurol*. 2015;6:110. doi:10.3389/fneur.2015.00110

11. Moser RS, Iverson GL, Echemendia RJ, et al. Neuropsychological evaluation in the diagnosis and management of sports-related concussion. *Arch Clin Neuropsychol*. Nov 2007;22(8):909-16. doi:10.1016/j.acn.2007.09.004

12. Langlois JA, Rutland-Brown W, Wald MM. The epidemiology and impact of traumatic brain injury: a brief overview. *J Head Trauma Rehabil*. Sep-Oct 2006;21(5):375-8.

13. McCrory P. Traumatic brain injury: revisiting the AAN guidelines on sport-related concussion. *Nat Rev Neurol*. Jul 2013;9(7):361-2. doi:10.1038/nrneurol.2013.88

14. Zemek R, Barrowman N, Freedman SB, et al. Clinical Risk Score for Persistent Postconcussion Symptoms Among Children With Acute Concussion in the ED. *JAMA*. Mar 8 2016;315(10):1014-25. doi:10.1001/jama.2016.1203

15. Williams RM, Puetz TW, Giza CC, Broglio SP. Concussion recovery time among high school and collegiate athletes: a systematic review and meta-analysis. *Sports Med*. Jun 2015;45(6):893-903. doi:10.1007/s40279-015-0325-8

16. McCrory P, Meeuwisse W, Dvorak J, et al. Consensus statement on concussion in sport-the 5th international conference on concussion in sport held in Berlin, October 2016. *Br J Sports Med*. Apr 26 2017;doi:10.1136/bjsports-2017-097699

17. Giza CC, Hovda DA. The new neurometabolic cascade of concussion. *Neurosurgery*. Oct 2014;75 Suppl 4:S24-33. doi:10.1227/NEU.0000000000000505

18. Silverberg ND, Iverson GL. Is rest after concussion "the best medicine?": recommendations for activity resumption following concussion in athletes, civilians, and military service members. *J Head Trauma Rehabil*. Jul-Aug 2013;28(4):250-9. doi:10.1097/HTR.0b013e31825ad658

19. Leddy J, Hinds A, Sirica D, Willer B. The Role of Controlled Exercise in Concussion Management. *PM R*. Mar 2016;8(3 Suppl):S91-S100. doi:10.1016/j.pmrj.2015.10.017

20. Cotman CW, Berchtold NC. Exercise: a behavioral intervention to enhance brain health and plasticity. *Trends Neurosci*. Jun 2002;25(6):295-301.

21. Krogh J, Nordentoft M, Sterne JA, Lawlor DA. The effect of exercise in clinically depressed adults: systematic review and meta-analysis of randomized controlled trials. *J Clin Psychiatry*. Apr 2011;72(4):529-38. doi:10.4088/JCP.08r04913blu

22. Neeper SA, Gomez-Pinilla F, Choi J, Cotman CW. Physical activity increases mRNA for brain-derived neurotrophic factor and nerve growth factor in rat brain. *Brain Res*. Jul 8 1996;726(1-2):49-56.

23. White PD, Goldsmith KA, Johnson AL, et al. Comparison of adaptive pacing therapy, cognitive behaviour therapy, graded exercise therapy, and specialist medical care for chronic fatigue syndrome (PACE): a randomised trial. *Lancet*. Mar 5 2011;377(9768):823-36. doi:10.1016/S0140-6736(11)60096-2

24. Wipfli BM, Rethorst CD, Landers DM. The anxiolytic effects of exercise: a meta-analysis of randomized trials and dose-response analysis. *J Sport Exerc Psychol*. Aug 2008;30(4):392-410.

25. Abaji JP, Curnier D, Moore RD, Ellemberg D. Persisting Effects of Concussion on Heart Rate Variability during Physical Exertion. *J Neurotrauma*. May 1 2016;33(9):811-7. doi:10.1089/neu.2015.3989

26. Griesbach GS, Gomez-Pinilla F, Hovda DA. The upregulation of plasticity-related proteins following TBI is disrupted with acute voluntary exercise. *Brain Res*. Aug 6 2004;1016(2):154-62. doi:10.1016/j.brainres.2004.04.079

27. Griesbach GS, Hovda DA, Gomez-Pinilla F. Exercise-induced improvement in cognitive performance after traumatic brain injury in rats is dependent on BDNF activation. *Brain Res*. Sep 8 2009;1288:105-15. doi:10.1016/j.brainres.2009.06.045

28. Griesbach GS, Hovda DA, Molteni R, Wu A, Gomez-Pinilla F. Voluntary exercise following traumatic brain injury: brain-derived neurotrophic factor upregulation and recovery of function. *Neuroscience*. 2004;125(1):129-39. doi:10.1016/j.neuroscience.2004.01.030

29. Itoh T, Imano M, Nishida S, et al. Exercise increases neural stem cell proliferation surrounding the area of damage following rat traumatic brain injury. *J Neural Transm (Vienna)*. Feb 2011;118(2):193-202. doi:10.1007/s00702-010-0495-3

30. La Fountaine MF, Gossett JD, De Meersman RE, Bauman WA. Increased QT interval variability in 3 recently concussed athletes: an exploratory observation. *J Athl Train*. May-Jun 2011;46(3):230-3.

31. Willer B, Leddy JJ. Management of concussion and post-concussion syndrome. *Curr Treat Options Neurol*. Sep 2006;8(5):415-26.

32. Gomez-Pinilla F, Dao L, So V. Physical exercise induces FGF-2 and its mRNA in the hippocampus. *Brain Res*. Aug 1 1997;764(1-2):1-8.

33. Gomez-Pinilla F, Ying Z, Opazo P, Roy RR, Edgerton VR. Differential regulation by exercise of BDNF and NT-3 in rat spinal cord and skeletal muscle. *Eur J Neurosci*. Mar 2001;13(6):1078-84.

34. Gomez-Pinilla F, Ying Z, Roy RR, Molteni R, Edgerton VR. Voluntary exercise induces a BDNF-mediated mechanism that promotes neuroplasticity. *J Neurophysiol*. Nov 2002;88(5):2187-95. doi:10.1152/jn.00152.2002

35. Kim DH, Ko IG, Kim BK, et al. Treadmill exercise inhibits traumatic brain injury-induced hippocampal apoptosis. *Physiol Behav*. Dec 2 2010;101(5):660-5. doi:10.1016/j.physbeh.2010.09.021

36. Seo TB, Kim BK, Ko IG, et al. Effect of treadmill exercise on Purkinje cell loss and astrocytic reaction in the cerebellum after traumatic brain injury. *Neurosci Lett*. Sep 13 2010;481(3):178-82. doi:10.1016/j.neulet.2010.06.087

37. Majerske CW, Mihalik JP, Ren D, et al. Concussion in sports: postconcussive activity levels, symptoms, and neurocognitive performance. *J Athl Train*. May-Jun 2008;43(3):265-74. doi:10.4085/1062-6050-43.3.265

38. Leddy JJ, Baker JG, Kozlowski K, Bisson L, Willer B. Reliability of a graded exercise test for assessing recovery from concussion. *Clin J Sport Med*. Mar 2011;21(2):89-94. doi:10.1097/JSM.0b013e3181fdc721

39. Leddy JJ, Kozlowski K, Donnelly JP, Pendergast DR, Epstein LH, Willer B. A preliminary study of subsymptom threshold exercise training for refractory post-concussion syndrome. *Clin J Sport Med*. Jan 2010;20(1):21-7. doi:10.1097/JSM.0b013e3181c6c22c

40. Leddy JJ, Willer B. Use of graded exercise testing in concussion and return-to-activity management. *Curr Sports Med Rep*. Nov-Dec 2013;12(6):370-6. doi:10.1249/JSR.0000000000000008

41. Harmon KG. Football concussion rates across school levels. *J Pediatr*. Jan 2016;168:253-4. doi:10.1016/j.jpeds.2015.10.071

42. Lincoln AE, Caswell SV, Almquist JL, Dunn RE, Norris JB, Hinton RY. Trends in concussion incidence in high school sports: a prospective 11-year study. *Am J Sports Med*. May 2011;39(5):958-63. doi:10.1177/0363546510392326

43. Chiu CC, Liao YE, Yang LY, et al. Neuroinflammation in animal models of traumatic brain injury. *J Neurosci Methods*. Jul 2 2016;doi:10.1016/j.jneumeth.2016.06.018

44. Gardner AJ, Zafonte R. Neuroepidemiology of traumatic brain injury. *Handb Clin Neurol*. 2016;138:207-23. doi:10.1016/B978-0-12-802973-2.00012-4

45. Shultz SR, Bao F, Omana V, Chiu C, Brown A, Cain DP. Repeated mild lateral fluid percussion brain injury in the rat causes cumulative long-term behavioral impairments, neuroinflammation, and cortical loss in an animal model of repeated concussion. *J Neurotrauma*. Jan 20 2012;29(2):281-94. doi:10.1089/neu.2011.2123

46. Hanisch UK, Kettenmann H. Microglia: active sensor and versatile effector cells in the normal and pathologic brain. *Nat Neurosci*. Nov 2007;10(11):1387-94. doi:10.1038/nn1997

47. Kumar A, Loane DJ. Neuroinflammation after traumatic brain injury: opportunities for therapeutic intervention. *Brain Behav Immun*. Nov 2012;26(8):1191-201. doi:10.1016/j.bbi.2012.06.008

48. Di Battista AP, Rhind SG, Baker AJ. Application of blood-based biomarkers in human mild traumatic brain injury. *Front Neurol*. 2013;4:44. doi:10.3389/fneur.2013.00044

49. Barkhoudarian G, Hovda DA, Giza CC. The Molecular Pathophysiology of Concussive Brain Injury - an Update. *Phys Med Rehabil Clin N Am*. May 2016;27(2):373-93. doi:10.1016/j.pmr.2016.01.003

50. Bergsneider M, Hovda DA, Lee SM, et al. Dissociation of cerebral glucose metabolism and level of consciousness during the period of metabolic depression following human traumatic brain injury. *J Neurotrauma*. May 2000;17(5):389-401. doi:10.1089/neu.2000.17.389

51. Bergsneider M, Hovda DA, Shalmon E, et al. Cerebral hyperglycolysis following severe traumatic brain injury in humans: a positron emission tomography study. *J Neurosurg*. Feb 1997;86(2):241-51. doi:10.3171/jns.1997.86.2.0241

52. Jalloh I, Carpenter KL, Grice P, et al. Glycolysis and the pentose phosphate pathway after human traumatic brain injury: microdialysis studies using 1,2-(13)C2 glucose. *J Cereb Blood Flow Metab*. Jan 2015;35(1):111-20. doi:10.1038/jcbfm.2014.177

53. Jalloh I, Carpenter KL, Helmy A, Carpenter TA, Menon DK, Hutchinson PJ. Glucose metabolism following human traumatic brain injury: methods of assessment and pathophysiological findings. *Metab Brain Dis*. Jun 2015;30(3):615-32. doi:10.1007/s11011-014-9628-y

54. Gall B, Parkhouse W, Goodman D. Heart rate variability of recently concussed athletes at rest and exercise. *Med Sci Sports Exerc*. Aug 2004;36(8):1269-74.

55. Ingebrigtsen T, Romner B, Kock-Jensen C. Scandinavian guidelines for initial management of minimal, mild, and moderate head injuries. The Scandinavian Neurotrauma Committee. *J Trauma*. Apr 2000;48(4):760-6.

56. McCrory P, Meeuwisse W, Johnston K, et al. Consensus Statement on Concussion in Sport: the 3rd International Conference on Concussion in Sport held in Zurich, November 2008. *Br J Sports Med*. May 2009;43 Suppl 1:i76-90. doi:10.1136/bjsm.2009.058248

57. Carson JD, Lawrence DW, Kraft SA, et al. Premature return to play and return to learn after a sport-related concussion: physician's chart review. *Can Fam Physician*. Jun 2014;60(6):e310, e312-5.

58. McLean SA, Clauw DJ. Predicting chronic symptoms after an acute "stressor"--lessons learned from 3 medical conditions. *Med Hypotheses*. 2004;63(4):653-8. doi:10.1016/j.mehy.2004.03.022

59. Winkelman C. Bed rest in health and critical illness: a body systems approach. *AACN Adv Crit Care*. Jul-Sep 2009;20(3):254-66. doi:10.1097/NCI.0b013e3181ac838d

60. Bernhardt J, Dewey H, Thrift A, Collier J, Donnan G. A very early rehabilitation trial for stroke (AVERT): phase II safety and feasibility. *Stroke*. Feb 2008;39(2):390-6. doi:10.1161/STROKEAHA.107.492363

61. Albert SJ, Kesselring J. Neurorehabilitation of stroke. *J Neurol*. May 2012;259(5):817-32. doi:10.1007/s00415-011-6247-y

62. Schnabel M, Ferrari R, Vassiliou T, Kaluza G. Randomised, controlled outcome study of active mobilisation compared with collar therapy for whiplash injury. *Emerg Med J*. May 2004;21(3):306-10.

63. Soderlund A, Olerud C, Lindberg P. Acute whiplash-associated disorders (WAD): the effects of early mobilization and prognostic factors in long-term symptomatology. *Clin Rehabil*. Oct 2000;14(5):457-67.

64. Waddell G, Feder G, Lewis M. Systematic reviews of bed rest and advice to stay active for acute low back pain. *Br J Gen Pract*. Oct 1997;47(423):647-52.

65. Dahm KT, Brurberg KG, Jamtvedt G, Hagen KB. Advice to rest in bed versus advice to stay active for acute low-back pain and sciatica. *Cochrane Database Syst Rev*. 2010;(6):CD007612. doi:10.1002/14651858.CD007612.pub2

66. Jacotte-Simancas A, Costa-Miserachs D, Torras-Garcia M, Coll-Andreu M, Portell-Cortes I. Effect of voluntary physical exercise and post-training epinephrine on acquisition of a spatial task in the barnes maze. *Behav Brain Res*. Jun 15 2013;247:178-81. doi:10.1016/j.bbr.2013.03.038

67. Itoh T, Imano M, Nishida S, et al. Exercise inhibits neuronal apoptosis and improves cerebral function following rat traumatic brain injury. *J Neural Transm (Vienna)*. Sep 2011;118(9):1263-72. doi:10.1007/s00702-011-0629-2

68. Gagnon I, Galli C, Friedman D, Grilli L, Iverson GL. Active rehabilitation for children who are slow to recover following sport-related concussion. *Brain Inj*. Nov 2009;23(12):956-64. doi:10.3109/02699050903373477

69. Colcombe SJ, Kramer AF, McAuley E, Erickson KI, Scalf P. Neurocognitive aging and cardiovascular fitness: recent findings and future directions. *J Mol Neurosci*. 2004;24(1):9-14. doi:10.1385/JMN:24:1:009

70. Erickson KI, Voss MW, Prakash RS, et al. Exercise training increases size of hippocampus and improves memory. *Proc Natl Acad Sci U S A*. Feb 15 2011;108(7):3017-22. doi:10.1073/pnas.1015950108

71. Murrell CJ, Cotter JD, Thomas KN, Lucas SJ, Williams MJ, Ainslie PN. Cerebral blood flow and cerebrovascular reactivity at rest and during sub-maximal exercise: effect of age and 12-week exercise training. *Age (Dordr)*. Jun 2013;35(3):905-20. doi:10.1007/s11357-012-9414-x

72. Gosselin N, Saluja RS, Chen JK, Bottari C, Johnston K, Ptito A. Brain functions after sports-related concussion: insights from event-related potentials and functional MRI. *Phys Sportsmed*. Oct 2010;38(3):27-37. doi:10.3810/psm.2010.10.1805

73. Keightley ML, Saluja RS, Chen JK, et al. A functional magnetic resonance imaging study of working memory in youth after sports-related concussion: is it still working? *J Neurotrauma*. Mar 1 2014;31(5):437-51. doi:10.1089/neu.2013.3052

74. Gagnon I, Grilli L, Friedman D, Iverson GL. A pilot study of active rehabilitation for adolescents who are slow to recover from sport-related concussion. *Scand J Med Sci Sports*. Mar 2016;26(3):299-306. doi:10.1111/sms.12441

75. Broglio SP, Cantu RC, Gioia GA, et al. National Athletic Trainers' Association position statement: management of sport concussion. *J Athl Train*. Mar-Apr 2014;49(2):245-65. doi:10.4085/1062-6050-49.1.07

76. Baillargeon A, Lassonde M, Leclerc S, Ellemberg D. Neuropsychological and neurophysiological assessment of sport concussion in children, adolescents and adults. *Brain Inj*. 2012;26(3):211-20. doi:10.3109/02699052.2012.654590

77. Alla S, Sullivan SJ, McCrory P. Defining asymptomatic status following sports concussion: fact or fallacy? *Br J Sports Med*. Jun 2012;46(8):562-9. doi:10.1136/bjsm.2010.081299

78. Lawrence DW, Richards D, Comper P, Hutchison MG. Earlier time to aerobic exercise is associated with faster recovery following acute sport concussion. *PLoS One*. 2018;13(4):e0196062. doi:10.1371/journal.pone.0196062

79. Csenge B. *Recovery from Concussion in a Cohort of Male High School Students*. University of Toronto; 2016.

# 17.0 Appendix

**David L. MacIntosh Sport Medicine Clinic**
