# Supplementary figures and images for: Randomized controlled trial of early aerobic exercise following sport-related concussion: Progressive percentage of age-predicted maximal heart rate versus usual care
Source: PLoS One. 2022 Dec 22;17(12):e0276336. doi: 10.1371/journal.pone.0276336 (PMC9778585; doi:10.1371/journal.pone.0276336)

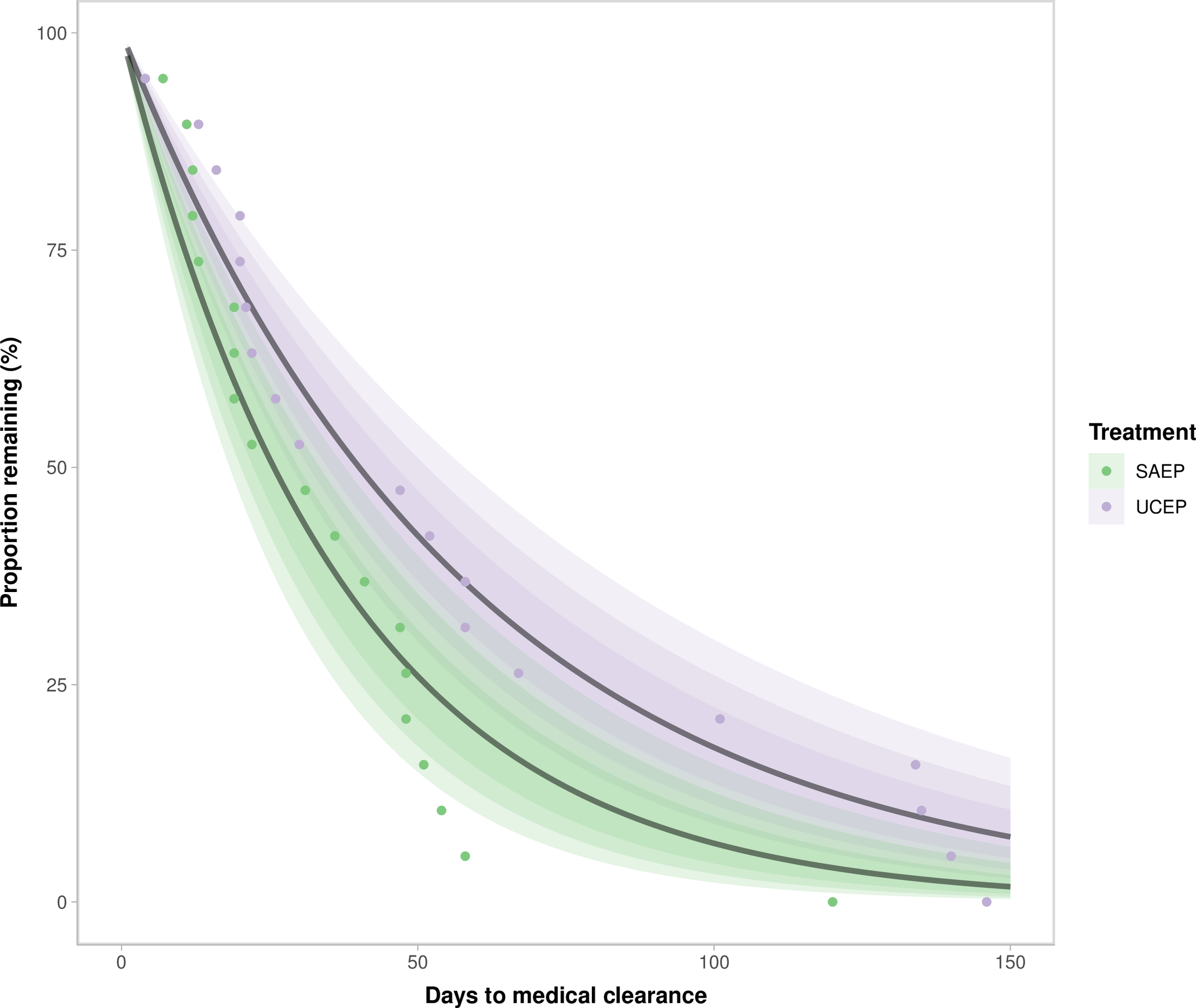

Supplement: S1 Fig — Exponential model for days to return to play overlaid on the complementary cumulative distribution of the raw data. (TIF) [file pone.0276336.s004.tif]

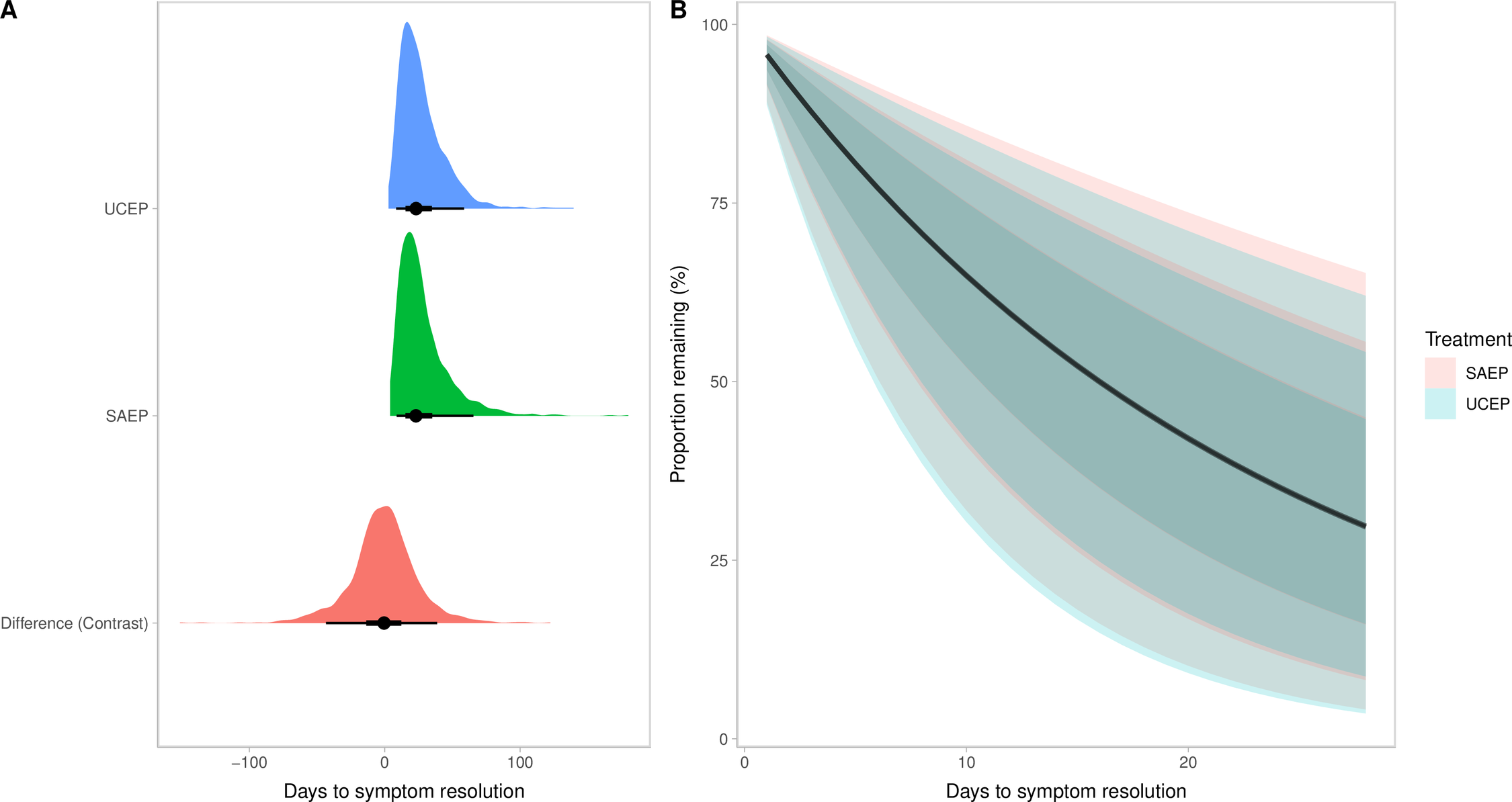

Supplement: S2 Fig — Prior simulations for exponential models evaluating days to asymptomatic status and days to medical clearance in the structured aerobic exercise prescription (SAEP) vs. usual care exercise prescription (UCEP) groups using a gaussian prior of the form α ~ normal(3.15, 0.6). Simulations results yielded an average time to asymptomatic status/return to play of ~28 days in both the SAEP and UCEP groups, with a standard deviation (SD) of ~18 days, and 90% compatibility interval (CI) of ~ 8.5–65 days. The mean difference between groups (contrast) was ~0, with a SD of ~25 days and 90% CI of ~ -40–40 days. (TIF) [file pone.0276336.s005.tif]

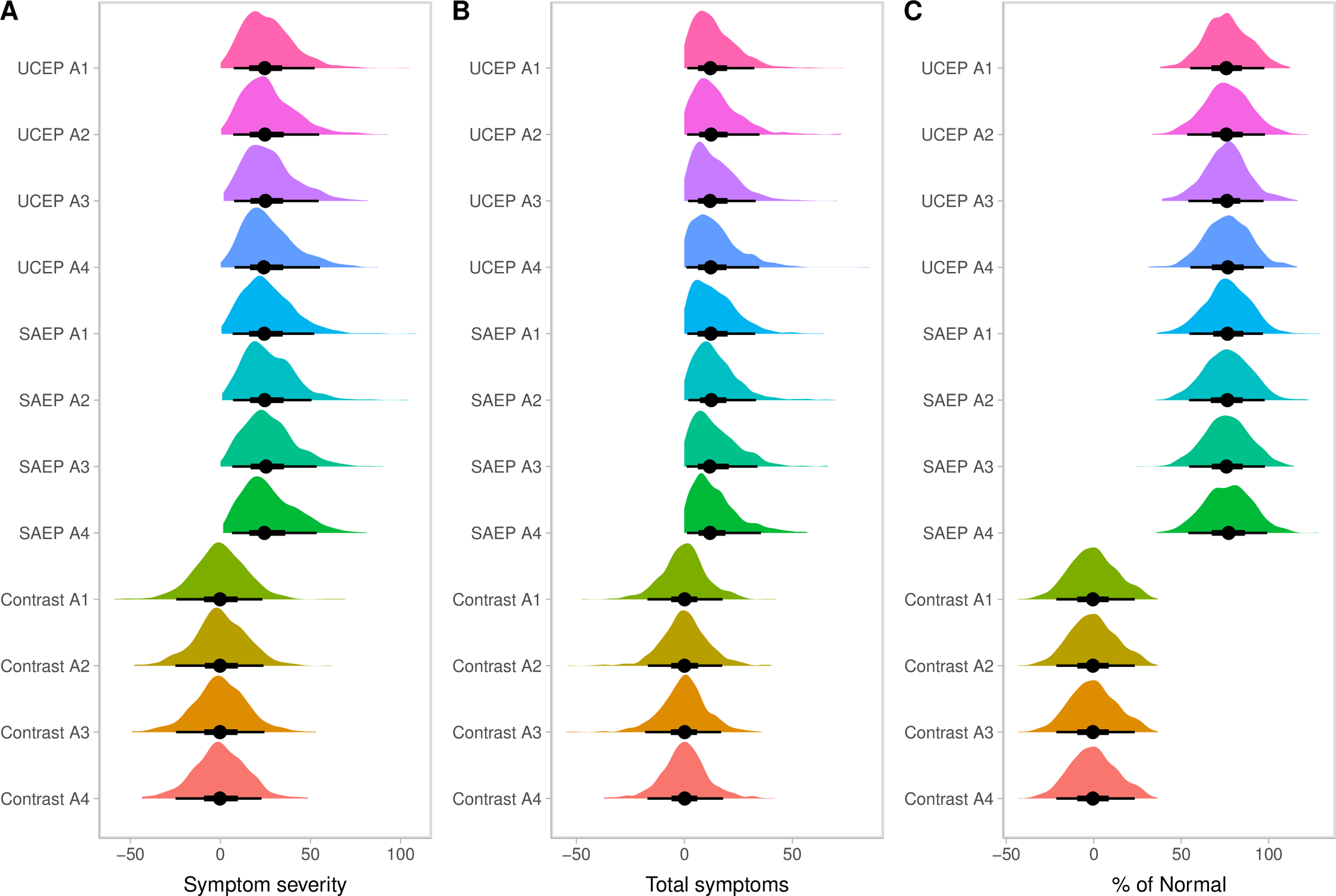

Supplement: S3 Fig — Histograms showing prior simulations for linear regression models evaluating A) Symptom Severity, B) Total Symptoms, and C) “% of Normal” at each of the four assessments of the trial (Assessments 1–4), as well as prior simulation contrasts at each session for the structured aerobic exercise prescription (SAEP) and usual care exercise prescription (UCEP) groups. (TIF) [file pone.0276336.s006.tif]

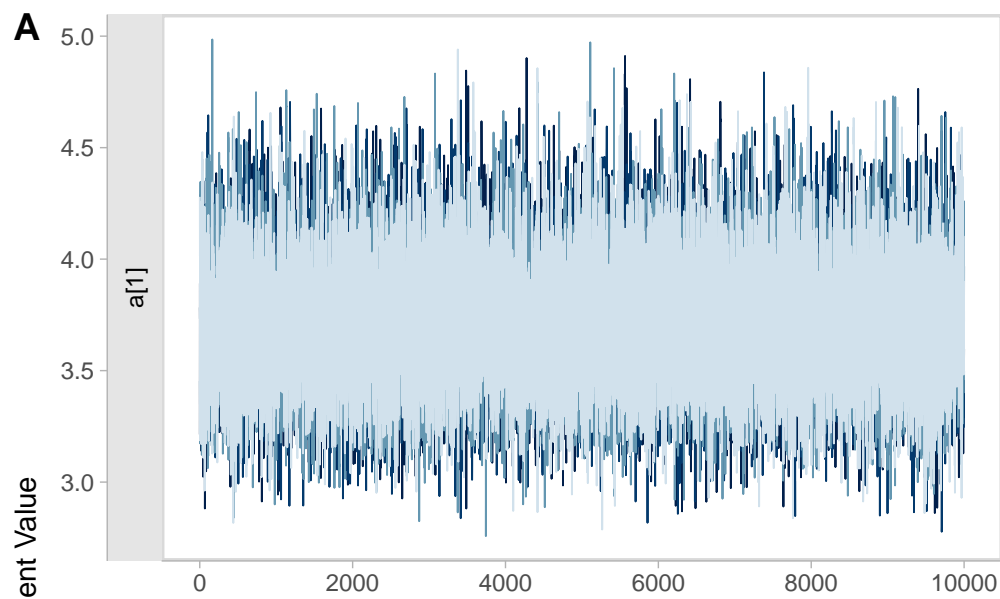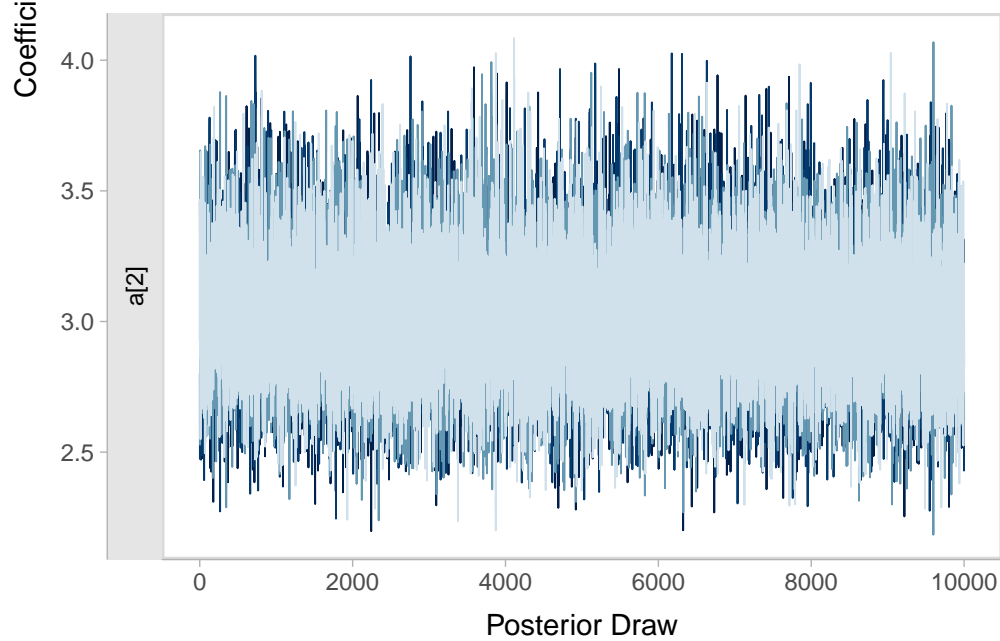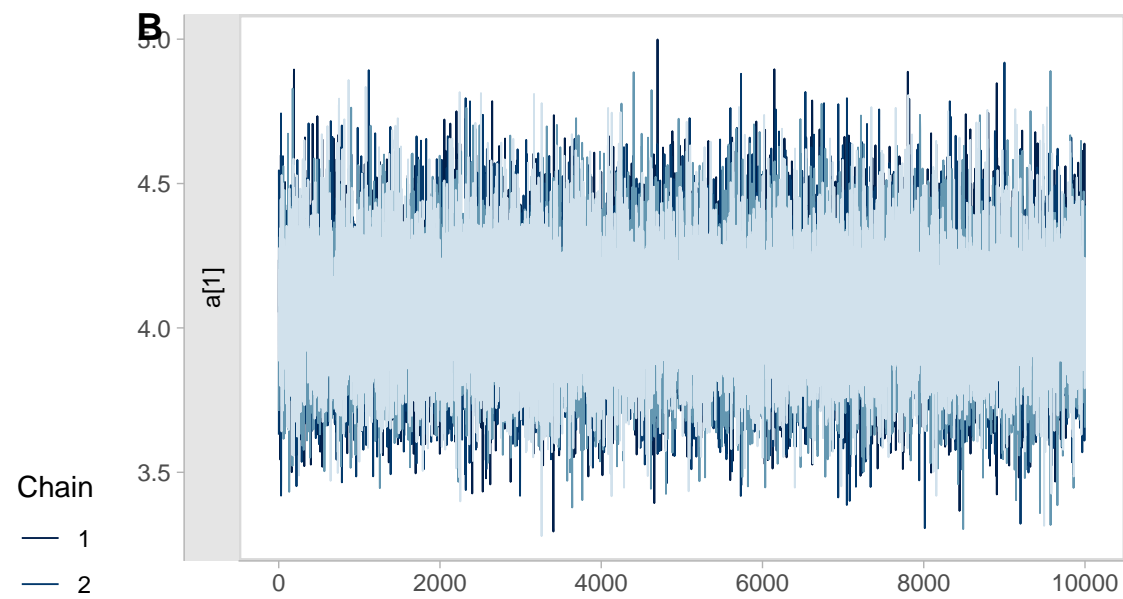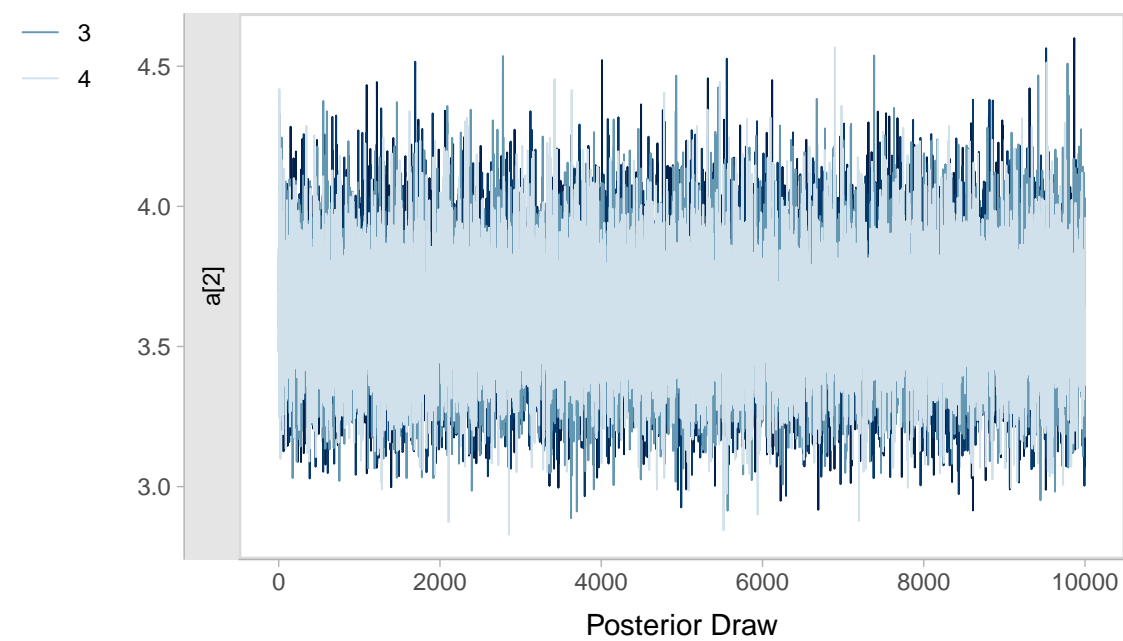

Chain

- 1
- 2
- 3
- 4

Chain

- 1
- 2
- 3
- 4

Supplement: S4 Fig — Traceplots for exponential survival curve models: (A) days to asymptomatic status, and (B) days to medical clearance. Plots are stratified by varying intercepts for usual care exercise prescription (UCEP) (a[1]) and structured aerobic exercise prescription groups (SAEP) (a[2]). Four chains were run at 20000 iterations per chain. All Gelman-Rubin values were <1.01. (PDF) [file pone.0276336.s007.pdf]
